# Anti-CD47 Antibody As a Targeted Therapeutic Agent for Human Lung Cancer and Cancer Stem Cells

**DOI:** 10.3389/fimmu.2017.00404

**Published:** 2017-04-21

**Authors:** Liang Liu, Lin Zhang, Lin Yang, Hui Li, Runmei Li, Jinpu Yu, Lili Yang, Feng Wei, Cihui Yan, Qian Sun, Hua Zhao, Fan Yang, Hao Jin, Jian Wang, Shizhen Emily Wang, Xiubao Ren

**Affiliations:** ^1^Department of Immunology, Tianjin Medical University Cancer Institute and Hospital, Tianjin, China; ^2^Department of Biotherapy, Tianjin Medical University Cancer Institute and Hospital, Tianjin, China; ^3^National Clinical Research Center of Cancer, Tianjin, China; ^4^Key Laboratory of Cancer Immunology and Biotherapy, Tianjin, China; ^5^State Key Laboratory of Experimental Hematology, Peking Union Medical College, Institute of Hematology and Blood Diseases Hospital, Chinese Academy of Medical Sciences, Tianjin, China; ^6^Department of Pathology, University of California, San Diego, CA, USA

**Keywords:** CD47, antibody, therapeutic agent, human lung cancer, cancer stem cells

## Abstract

Accumulating evidence indicates that a small subset of cancer cells, termed the tumor-initiating cells or cancer stem cells (CSCs), construct a reservoir of self-sustaining cancer cells with the characteristic ability to self-renew and maintain the tumor mass. The CSCs play an important role in the tumor initiation, development, relapse, metastasis, and the ineffectiveness of conventional cancer therapies. CD47 is a ligand for signal-regulatory protein-α expressed on phagocytic cells and functions to inhibit phagocytosis. This study was to explore if the expression of CD47 is the mechanism used by lung cancer cells, especially CSCs, to escape phagocytosis *in vitro* and *in vivo*. Here, we selected CD133 as the marker for lung CSCs according to previous reports. We analyzed lung cancer and matched adjacent normal (non-tumor) tissue and revealed that CD47 is overexpressed on lung cancer cells, especially on lung CSCs. The mRNA expression levels of *CD47* and *CD133* correlated with a decreased probability of survival for multiple types of lung cancer. Blocking CD47 function with anti-CD47 antibodies enabled macrophage phagocytosis of lung cancer cells and lung CSCs. Anti-CD47 antibodies inhibited tumor growth in immunodeficient mouse xenotransplantation models established with lung cancer cells or lung CSCs and improved survival in tumor-bearing animals. These data indicate that CD47 is a valid target for cancer therapies, especially for anti-CSC therapies.

## Introduction

Tumors are organized in a cellular hierarchy maintained by a small pool of self-renewing cancer stem cells (CSCs) or tumor-initiating cells (TICs), which must be removed to eliminate the tumor, according to the TIC or CSC model (1, 2). Candidate TICs have been prospectively isolated from a variety of solid tumors, including lung (3–6), breast (7, 8), brain (9), colorectal (10, 11), head and neck (12), pancreatic (13), prostate (14), and melanoma (15), based primarily on the expression of CD44, CD133, ALDH, and ABCB5 that have been recognized as markers for CSC enrichment. For the development of CSC-targeted therapies, it is necessary to identify molecules and pathways that are preferentially expressed in CSCs and critical for cancer pathogenesis and stemness.

CD47 is a widely expressed transmembrane protein with numerous functions (16). It functions as a ligand for signal-regulatory protein-α (SIRPα), a protein expressed on phagocytes, such as macrophages and dendritic cells (17). SIRPα initiates a signaling cascade through binding CD47, which results in the inhibition of phagocytosis (16). Blood cells, such as red blood cells, platelets, and lymphocytes, require CD47 expression on their membranes to protect themselves from rapid elimination by splenic macrophages (18–20). CD47 is upregulated in the migrating hematopoietic stem cells (HSCs), which protect themselves from phagocytosis by phagocytes as they pass through phagocyte-lined sinusoids (21). As such, CD47 expression levels predict the probability of HSCs to be phagocytosed during the circulation (21). CD47 is expressed at even higher levels on leukemia stem cells (LSCs) than their normal counterparts. Higher expression levels of CD47 on human LSCs contribute to pathogenesis by inhibiting their phagocytosis through the interaction of CD47 with an inhibitory receptor on phagocytes (22). Accumulating evidence suggests that CD47 expression on human solid tumor cells and especially CSCs is a common mechanism through which these cells protect themselves from phagocytosis, allowing tumor cell proliferation and metastasis (23–28).

This study was to explore whether the expression of CD47 is the mechanism used by lung cancer cells, especially CSCs, to escape phagocytosis *in vitro* and *in vivo*. We selected CD133 as a marker for lung CSCs (3–6). We show that CD47 is highly expressed on lung cancer cells and lung CSCs compared to its normal counterparts. Increased *CD47* and *CD133* expression levels in lung cancer patients correlated with a decreased probability of survival. Monoclonal antibodies targeting CD47 enabled the phagocytosis of patient-derived lung cancer cells and CSCs *in vitro* and inhibited the *in vivo* growth of xenografted tumors developed from patient-derived lung cancer cells or CSCs. These results indicate that CD47 is a critical regulator of innate immune surveillance and show that CD47 is a valid target for lung CSC therapies.

## Materials and Methods

### Cell Lines

The lung adenocarcinoma (AC) cell line A549 and lung squamous cell carcinoma (SCC) cell line NCI-H520 were obtained from the American Type Culture Collection. The LC3 and LC9 cell lines were generated from patients with small cell lung carcinoma (SCLC) and AC, respectively, by culturing bulk cells *in vitro* with IMDM supplemented with 10% human serum for 2 months.

### Human Samples

Tumor and matched adjacent normal (non-tumor) tissue specimens were defined by pathologists at Tianjin Medical University Cancer Institute and Hospital. Tumor specimens were cut to 1–2 mm^3^ masses and then enzymatically dissociated in Medium 199 containing collagenase III and DNase I (Sigma-Aldrich, St. Louis, MO, USA) at 37°C for 2–3 h, until single-cell suspension was obtained. Cells were then washed twice with PBS and filtered through a 70-µm filter.

### Flow Cytometry Analysis

For analysis of human lung cancer cell lines, primary tumor cells, and matched adjacent normal (non-tumor) cells, the following antibodies were used: CD45-APC, CD31-APC, CD47-Percp/Cy5 (BioLegend, San Diego, CA, USA) and ESA-FITC, CD133/1-PE (MiltenyiBiotec, Via Persicetana, Bologna, Italy). For analysis of mouse HSC in bone marrow, the following antibodies were used: Lin (V450 Mouse Lineage antibody Cocktail) (BD Bioscience, San Diego, CA, USA) and C-Kit-PE/Cy7, Sca-1-APC (BioLegend). Other antibodies include anti-mouse F4/80-PE/Cy7 and anti-human CD14-PE/Cy7 (Ebiosciences, San Diego, CA, USA). FACS analysis and cell sorting were performed on a BDFACSAria (Becton Dickinson) cell-sorting system under 20 psi with a 100-µm nozzle.

### Evaluation of Prognostic Value of CD47 and CD133 in Lung Cancer

Tianjin Medical University Cancer Institute and Hospital pathologists defined 317 patients tumor and 31 adjacent normal (non-tumor) tissue specimens. Total RNA of these tissues were provided by the National Clinical Research Center of Cancer of China. The mean of the 31 adjacent normal tissues RNA was regarded as the control RNA. The following primer sequences are used for PCR: CD47 cDNA F: ATC CGG TGG TAT GGA TGA GA, CD47 cDNA R: GGC AAT GAC GAA GGA GGT TAA, CD133 cDNA F: GCT TTG CAA TCT CCC TGT TG, CD133 cDNA R: TTG ATC CGG GTT CTT ACC TG. Real-time PCR was performed on ABI-9700. The definitions of overall survival (OS) and progression-free survival (PFS) were based on the RECIST. OS was calculated from the time of initiation therapy until death, and living patients were censored at the time of last contact. PFS was calculated from the time of initiation therapy until first progression, and patients alive and in a stable condition were censored at the time of last contact. The χ^2^ test and Fisher exact test were used for binary variable comparisons. The Mann–Whitney *U* test was used for median comparisons. The distributions of survival times and rates were estimated using the Kaplan–Meier method; the median survival times with 95% confidence intervals were reported. Associations between survival and potential prognostic factors were assessed using the log-rank test in a univariate analysis. The Cox proportional hazards model was undertaken in multivariable analyses by using the Forward-LR method with a significance level of 0.15 for entering and removing variables. In univariate evaluations of the prognostic impact of a continuous variable, the optimal cutoff was determined using the ROC method. A *P* value less than 0.05 using two-sided tests indicates significance. All calculations were performed using the SPSS 16.0 software.

### Preparation of Mouse and Human Macrophages

BALB/c mouse bone marrow mononuclear cells were harvested and grown in IMDM containing 10% FBS supplemented with 10 ng/mL recombinant murine macrophage colony-stimulating factor (Peprotech, Rocky Hill, NJ, USA) for 7–10 days to allow terminal differentiation of monocytes to macrophages. Human peripheral blood mononuclear cells were prepared from discarded normal blood from the Tianjin Medical University Cancer Institute and Hospital. Monocytes were isolated by adhering mononuclear cells to culture plates for 1 h at 37°C, after which non-adherent cells were removed by washing. The remaining cells were >95% CD14 and CD11b positive. Adherent cells were then incubated in IMDM plus 10% human serum for 7–10 days to allow terminal differentiation of monocytes to macrophages.

### *In Vitro* Phagocytosis Assay

Bone marrow-derived macrophages of BALB/c mice or peripheral blood-derived macrophages of patients were prepared and harvested by incubation in trypsin/EDTA (Gibco) for 5 min and gentle scraping. A total of 5 × 10^4^ macrophages were plated per well in a 24-well plate. Tumor cells were marked with 2.5 µM carboxyfluoresceinsuccinimidyl ester (CFSE) according to the manufacturer’s protocol (Invitrogen). Macrophages were incubated in serum-free IMDM for 4 h before adding 2 × 10^5^ CFSE-marked live tumor cells. The indicated antibodies (10 µg/mL) were added and incubated for 2 h at 37°C (26). After coincubation, wells were washed thoroughly with IMDM three times and subsequently imaged with fluorescence microscopy. The phagocytic index was calculated as the number of phagocytosed CFSE-positive cells per 100 macrophages. At least 200 macrophages were counted per well.

### Therapeutic Antibodies

The therapeutic antibodies and controls included anti-human CD47 B6H12.2 (Abcam, Cambridge, MA, USA); anti-human SIRPα/β antibody SE5A5; anti-mouse CD47 MIAP301 (Biolegend); mouse IgG1 isotype; rat IgG2 isotype; anti-HLA-A, B, and C (Ebiosciences); anti-mouse SIRPα; and P84 (BD Pharmingen).

### Generation of Luciferase-Positive Cell Lines and Luciferase Imaging Analysis

Lentivirus vector pUbi-MCS-LUC-IRES-puromycin was purchased from Shanghai (GENE, Shanghai, China). Lung cancer cell lines and primary lung cancer cells were transduced with the lentivirus and selected by puromycin following standard protocols and grown for several generations to ensure stability of the transgenes.

### *In Vivo* Precoating Engraftment Assay

Luciferase-expressing human lung cancer cell lines and primary lung cancer cells and their FACS-purified CSCs were precoated with 10 µg/mL IgG1 isotype control or anti-CD47 B6H12.2 antibody for half an hour *in vitro*. A total of 5 × 10^6^ precoated lung cancer cells or 1 × 10^4^ precoated lung CSCs were transplanted subcutaneously into NOD/SCID mice. All experiments involving mice were performed according to Tianjin Medical University Cancer Institute and Hospital animal guidelines. The tumor size in mice was measured by bioluminescence.

### *In Vivo* Antibody Treatment Xenograft Model

Luciferase-labeled A549 cells or LC3 primary lung cancer cells were injected subcutaneously at 5 × 10^6^ into the 6- to 8-week-old NOD/SCID mice. Those mice with luciferase-positive tumors after 5–6 weeks were given daily intraperitoneal injections of 400 µg mouse IgG1 control or anti-human CD47 B6H12.2 antibody for 4 weeks. Antibody treatment was then stopped, and mice were followed for survival analysis. Luciferase-labeled NCI-H520 CSCs or LC9 primary lung CSCs were transplanted subcutaneously at 1 × 10^4^ into the 6- to 8-week-old NOD/SCID mice. Those mice with luciferase-positive lung cancer after 9–10 weeks were given daily intraperitoneal injections of 400 µg mouse IgG1 control or anti-human CD47 B6H12.2 antibody for 4 weeks. Antibody treatment was then stopped, and mice were followed for survival analysis.

## Results

### CD47 Expression Is Increased on Lung Cancer Cells and Lung CSCs Compared to Their Normal Counterparts

**Figure 1:**
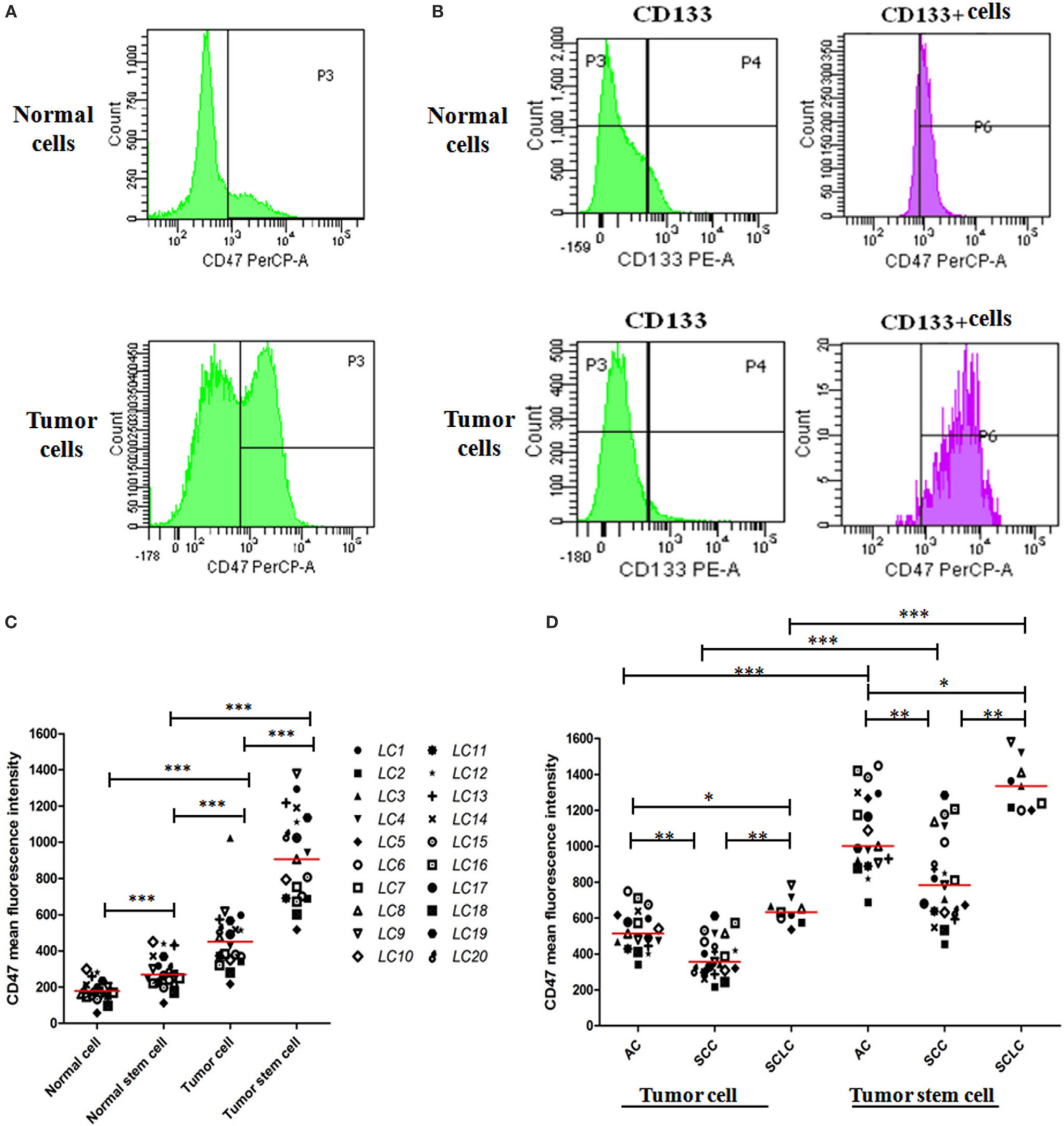
**CD47 expression is increased on lung cancer cells and lung cancer stem cells (CSCs) compared to their normal counterparts**. **(A)** Levels of CD47 expression were detected on tumor cells and matched adjacent normal cells (normal cells) by flow cytometry (FACS). Cells analyzed were CD45^−^, CD31^−^, DAPI^−^, and ESA^+^. **(B)** CD47 expression levels were detected on tumor stem cells CSCs and matched adjacent normal stem cells (normal stem cells) by FACS using the markers CD45^−^, CD31^−^, DAPI^−^, ESA^+^, and CD133^+^ to selected cells for analysis. **(C)** CD47 expression on normal cells, normal stem cells, tumor cells, and CSCs was determined by FACS. Mean fluorescence intensity was normalized for cell size. Each data point represents a different patient sample: adenocarcinoma (AC) = 9, squamous cell carcinoma (SCC) = 9, small cell lung carcinoma (SCLC) = 2. *P* values were calculated using the paired-samples *t*-test. **(D)** CD47 expression across lung cancer subtypes including AC (*n* = 19), SCC (*n* = 21), and SCLC (*n* = 9) was determined as in **(C)**. *P* values were calculated using the paired-samples *t*-test or the Two-independent samples test Mann–Whitney *U* model. *P < 0.05, **P < 0.01, and ***P < 0.001.

We evaluated CD47 expression on dissociated primary lung tumor cells (tumor cells) and matched adjacent normal cells (normal cells) from human patients by flow cytometry (FACS). CD45^−^, CD31^−^, DAPI^−^, and ESA^+^ were used as the markers to detect viable lung tumor cells and normal cells. CD47 expression was detected on lung tumor cells and normal cells (Figure 1A) and on lung CSCs (CD133^+^) and normal stem cells (CD133^+^) (Figure 1B). Tumor cells expressed higher levels of CD47 than normal cells (Figure 1C). In addition, CSCs expressed higher levels of CD47 than normal stem cells (Figure 1C). These samples included lung AC, lung SCC, and SCLC (Figure S1 in Supplementary Material). Across different lung cancer subtypes, we found varying levels of CD47 expression that was significantly different within each lung cancer subtype (Figure 1D).

### Increased *CD47* and *CD133* Expression Correlate with a Worse Clinical Prognosis

**Figure 2:**
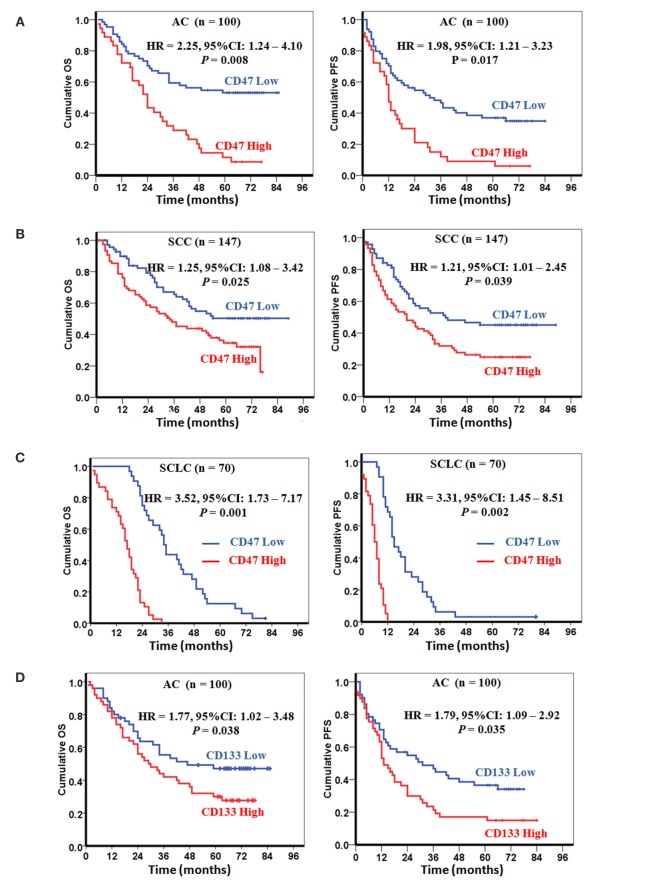

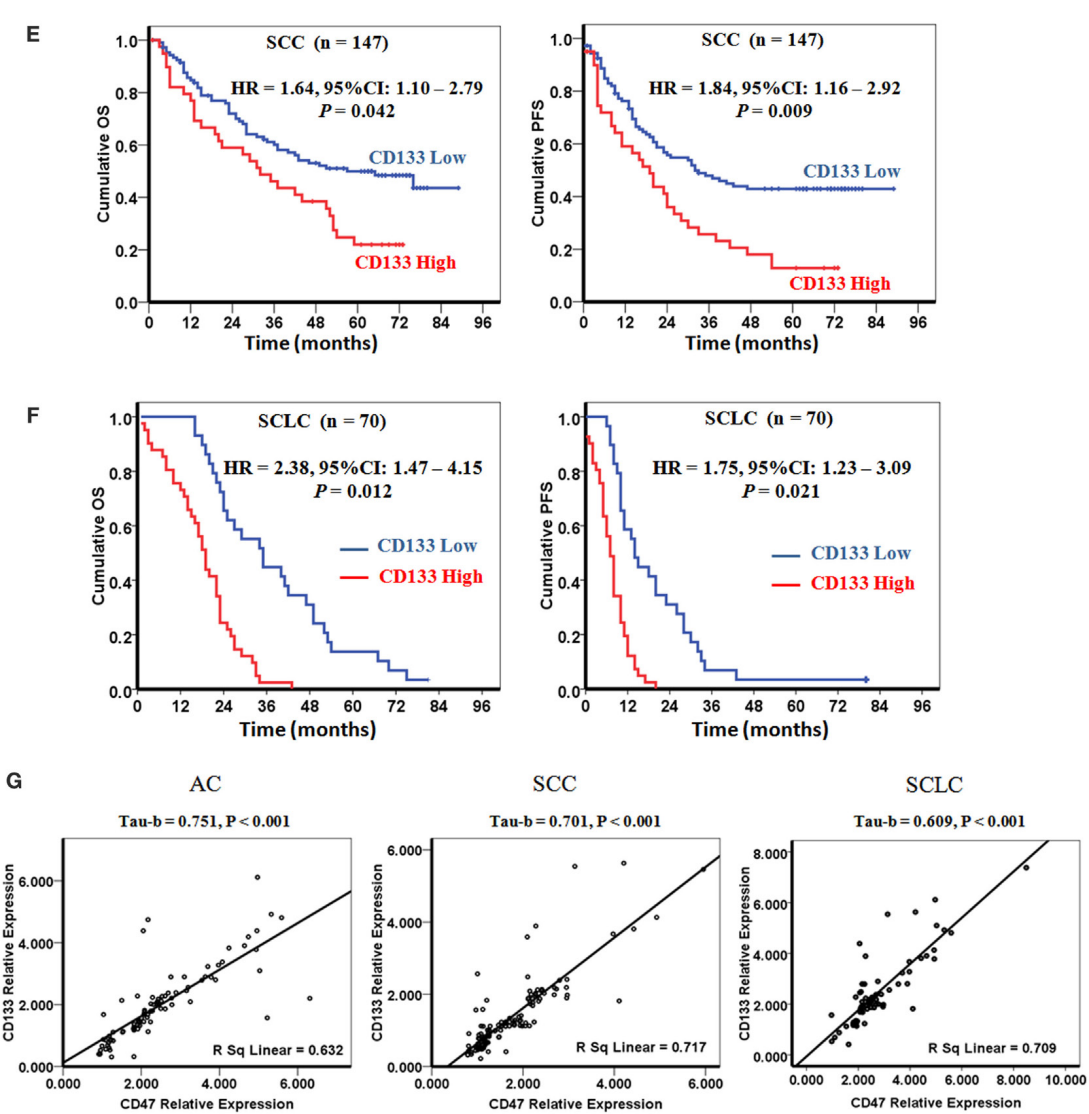
**Increased *CD47* and *CD133* expressions correlate with a worse clinical prognosis**. **(A–C)** *CD47* mRNA level is an independent prognostic factor in lung cancers. Increased levels of *CD47* mRNA were correlated with decreased probability of overall survival (OS) and progression-free survival (PFS) of patients with adenocarcinoma (AC) **(A)**, squamous cell carcinoma (SCC) **(B)**, and small cell lung carcinoma (SCLC) **(C)**. **(D–F)** *CD133* mRNA level is an independent prognostic factor in lung cancers. Prognostic impact of *CD133* mRNA level is shown as the correlation with OS and PFS of patients with AC **(D)**, SCC **(E)**, and SCLC **(F)**. *P* values were calculated using the Cox regression forward-LR model **(A–F)**. **(G)** *CD47* mRNA levels were positive correlation with the *CD133* mRNA levels in patients with AC, SCC, and SCLC. *P* values were calculated using the Bivariate Correlations Kendall’s tau-b model.

To determine whether *CD47* mRNA level serves as a prognostic factor in human lung cancers, we retrospectively analyzed gene expression data of 317 lung cancer patients including 100 AC (Table S1 in Supplementary Material), 147 SCC (Table S2 in Supplementary Material), and 70 SCLC (Table S3 in Supplementary Material). Distributions of relative *CD47* and *CD133* mRNA expression levels and clinical characteristics were shown in Tables S1–S3 in Supplementary Material. In a multivariable analysis, stratification of patients into “*CD47* high” and “*CD47* low” groups indicated that high *CD47* mRNA expression levels could decrease the OS and PFS in patients with AC (Figure 2A), SCC (Figure 2B), and SCLC (Figure 2C). We also detected *CD133* mRNA expression levels in the 317 lung cancer patients. In a multivariable analysis, a higher *CD133* mRNA expression level was associated with a decreased probability of OS and PFS in patients with AC (Figure 2D), SCC (Figure 2E), and SCLC (Figure 2F). The results of multivariable analysis in AC, SCC, and SCLC were shown in Tables S4–S6 in Supplementary Material. We extended our analysis to double-positive patients. The results show that *CD47* and *CD133* mRNA double higher expression levels can decrease the OS and PFS in patients with AC (Figure S2A in Supplementary Material), SCC (Figure S2B in Supplementary Material), and SCLC (Figure S2C in Supplementary Material). These results reveal that *CD47* and *CD133* expression levels may be independent prognostic factors in lung cancer patients. We further analyzed the expression correlation of *CD47* and *CD133* mRNA in the 317 lung cancer patients. Significant positive correlation was detected between *CD47* mRNA and *CD133* mRNA expression levels in AC, SCC, and SCLC patients (Figure 2G).

### Anti-CD47-Blocking Antibodies Enable Phagocytosis of Lung Cancer Cells and Lung CSCs by Macrophages *In Vitro*

**Figure 3:**
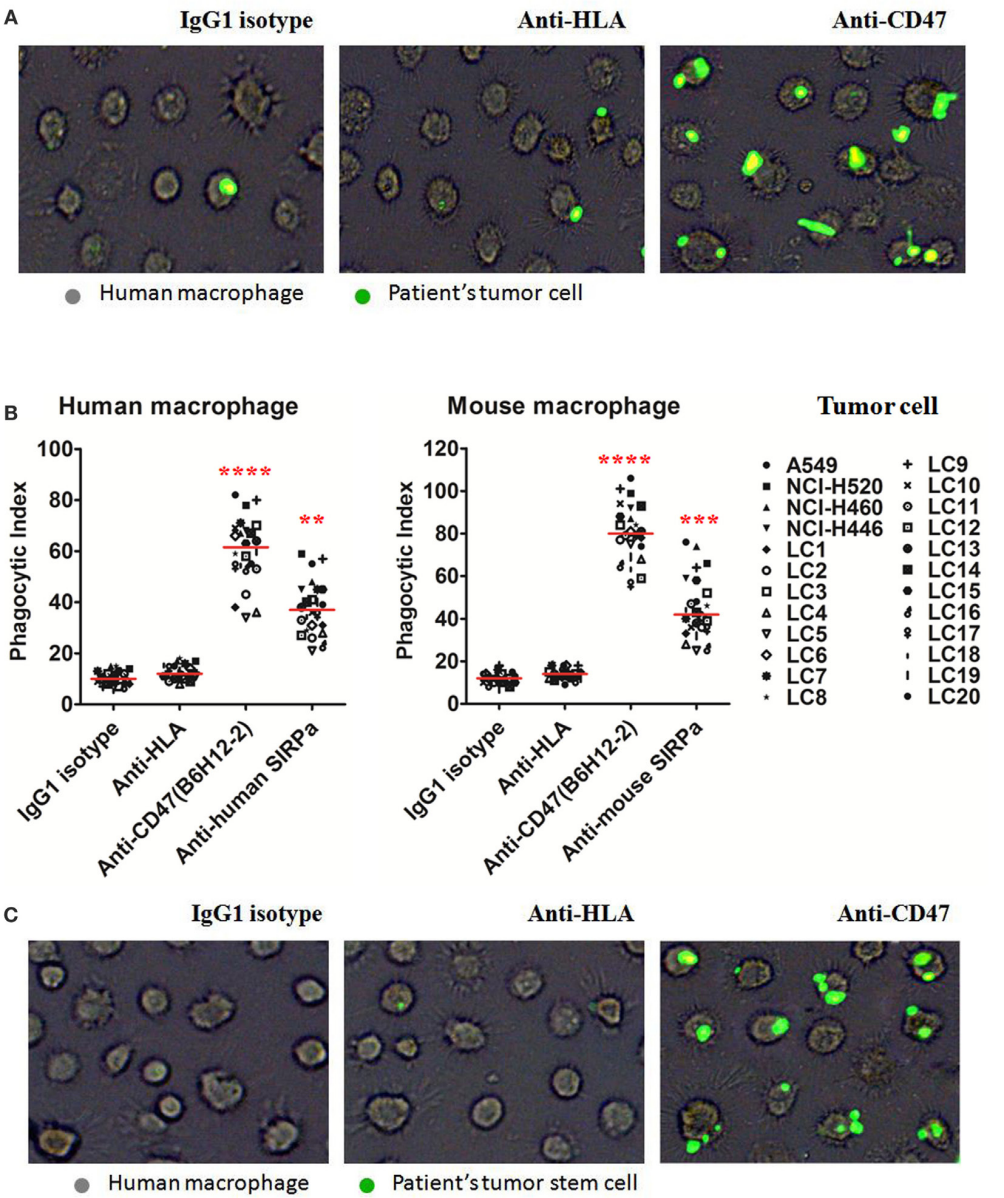

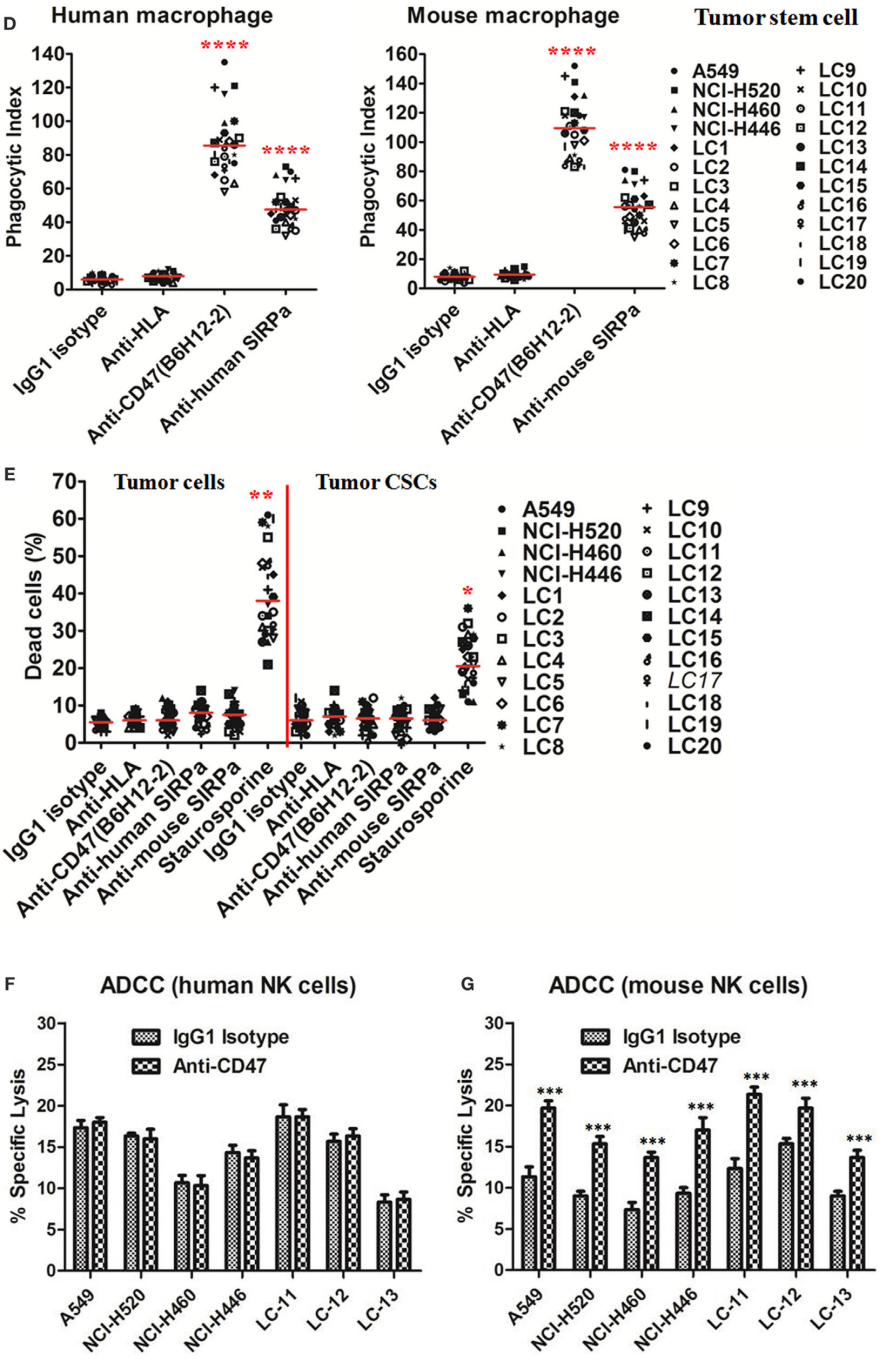
**Blocking antibodies against CD47 enable phagocytosis of lung cancer cells and lung cancer stem cells (CSCs) by macrophages *in vitro***. **(A,B)** Carboxyfluoresceinsuccinimidyl ester (CFSE)-labeled lung cancer cells were incubated with human macrophages or mouse macrophages and the indicated antibodies and examined by immunofluorescence microscopy to detect phagocytosis. **(A)** Images from a representative lung cancer sample are shown. **(B)** Phagocytic indices of primary human lung cancer cells and lung cancer cell lines were determined using human (left) and mouse (right panel) macrophages. **(C,D)** CFSE-labeled lung CSCs were incubated with human macrophages or mouse macrophages as well as the indicated antibodies and examined by immunofluorescence microscopy to detect phagocytosis. **(C)** Images from a representative lung CSCs sample are shown. **(D)** Phagocytic indices of primary human lung CSCs and lung cancer cell lines CSCs were determined using human (left) and mouse (right panel) macrophages. **(E)** Antibody-induced apoptosis was tested by incubating lung cancer cells or lung CSCs with the indicated antibodies or staurosporine without macrophages and assessing the percentage of apoptotic and dead cells (% annexin V and/or PI positive). **(F,G)** Chromium release assays measuring ADCC were performed in triplicate with human **(F)** and mouse **(G)** at an effector:target ratio of 20:1, and percent specific lysis is reported. Antibodies were incubated at 10 μg/mL. *P* values were calculated using the two-independent samples test Mann–Whitney *U* model. *P < 0.05, **P < 0.01, ***P < 0.001, and ****P < 0.0001.

We first tested the ability of anti-human CD47 antibody to enable phagocytosis of human lung cancer cell lines and primary lung cancer cells by human and mouse macrophages *in vitro*. Incubation of lung cancer cells in the presence of isotype-matched IgG1 control or anti-HLA antibody did not result in significant phagocytosis by human and mouse macrophages. In contrast, human and mouse macrophages could efficiently phagocytose lung cancer cells treatment with the anti-human CD47-blocking antibody B6H12.2 (Figures 3A,B). Disruption of the CD47–SIRPα interaction with anti-human or anti-mouse SIRPα antibody also resulted in significant phagocytosis of lung cancer cells (Figure 3B). Next, we repeated the *in vitro* phagocytosis assays with lung CSCs. Purified lung CSCs were isolated from lung cancer cell lines and primary lung cancer cells by FACS (Figure 1B). Incubation of purified lung CSCs in the presence of isotype-matched IgG1 control or anti-HLA antibody did not result in significant phagocytosis by human and mouse macrophages; however, lung CSCs treated with the anti-human CD47-blocking antibody B6H12.2 were significantly phagocytosed by human and mouse macrophages (Figures 3C,D). Disruption of the CD47–SIRPα interaction with anti-human or anti-mouse SIRPα antibody also resulted in significant phagocytosis of purified lung CSCs (Figure 3D). These results suggest that CD47 is a feasible therapy target and that anti-human CD47-blocking antibody may serve as an effective therapeutic agent to inhibit lung cancer growth by enabling macrophages to eliminate both CSCs and their differentiated progeny.

We further detected whether the anti-human CD47 antibody can influence the apoptosis or proliferation of lung cancer cells or lung CSCs. Lung cancer cells or lung CSCs were incubated with the indicated soluble antibodies in suspension for 8 h. Anti-human CD47 antibody did not increase the apoptosis of lung cancer cells or CSCs compared to IgG isotype or anti-HLA antibodies (Figure 3E; Figure S3A in Supplementary Material). Similar negative results were observed with anti-human SIRPα and anti-mouse SIRPα (Figure 3E). Then, lung cancer cells or lung CSCs were treated with the indicated soluble antibodies in adherence for 48 h. Anti-human CD47, anti-human SIRPα, or anti-mouse SIRPα has no effect on the proliferation of lung cancer cells (Figures S3B,C in Supplementary Material). We also investigated whether NK cells could mediate tumor elimination by anti-CD47 antibody *in vitro*. By utilizing human NK cells (CD3^−^CD56^+^CD7^+^) as effectors, anti-CD47 antibody did not induce increased ADCC of lung cancer cell line cells or primary patients tumor cells compared to IgG1 isotype control (Figure 3F). As anti-CD47 antibody (B6H12.2) is a mouse IgG1 isotype, we repeated these assays with mouse NK cells (CD3^−^DX5^+^). Anti-CD47 antibody caused increased ADCC of these cells compared to isotype control (Figure 3G). These data indicate that the mechanism of anti-CD47 antibody to inhibit cancer cells or CSC growth is unlikely to be that these antibodies induce apoptosis of lung cancer cells or CSCs that are then secondarily phagocytosed or directly inhibit proliferation of lung cancer cells.

### *Ex Vivo* Coating of Lung Cancer Cells and Lung CSCs with an Anti-CD47 Antibody Inhibits Tumor Engraftment

**Figure 4:**
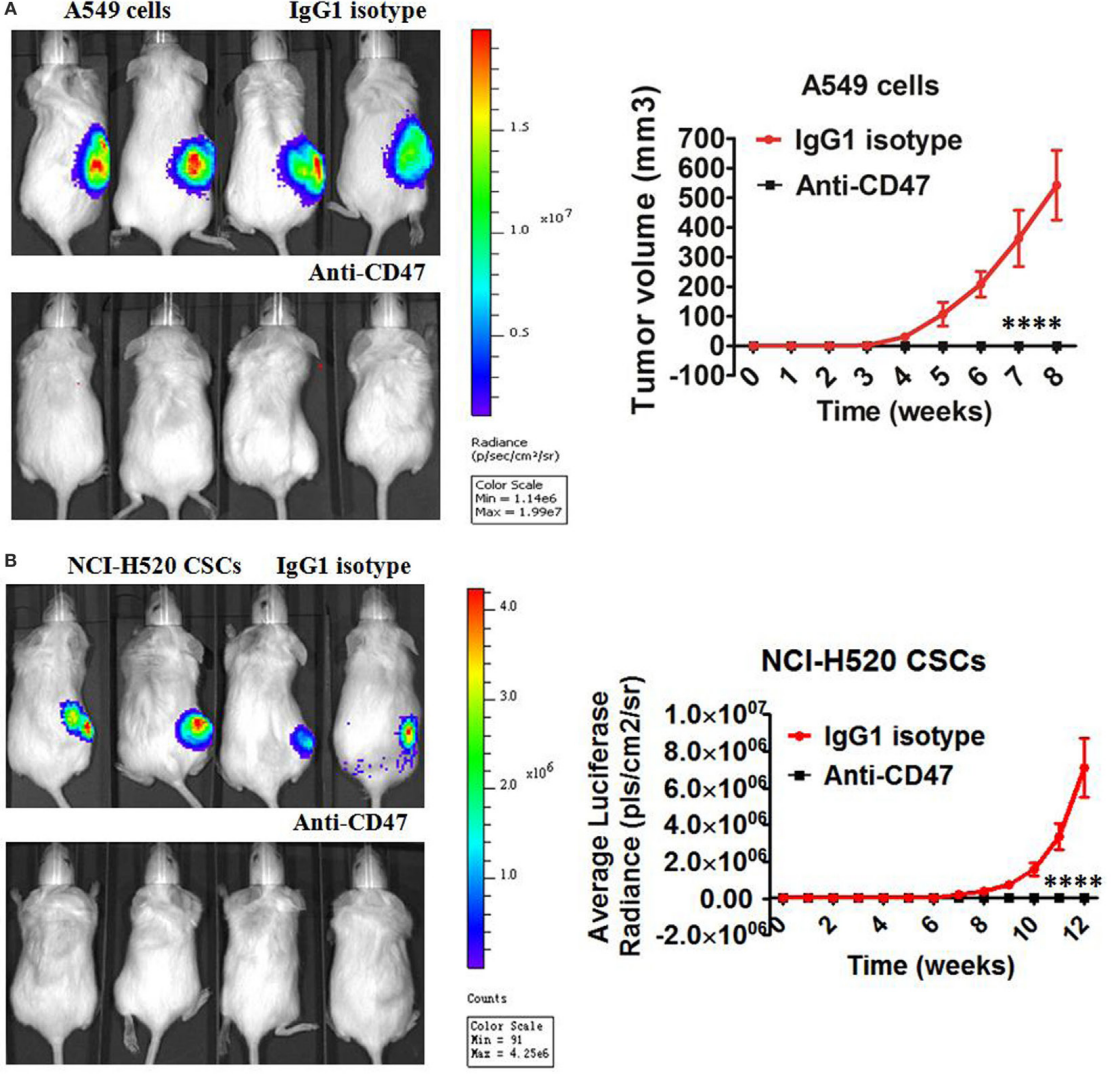

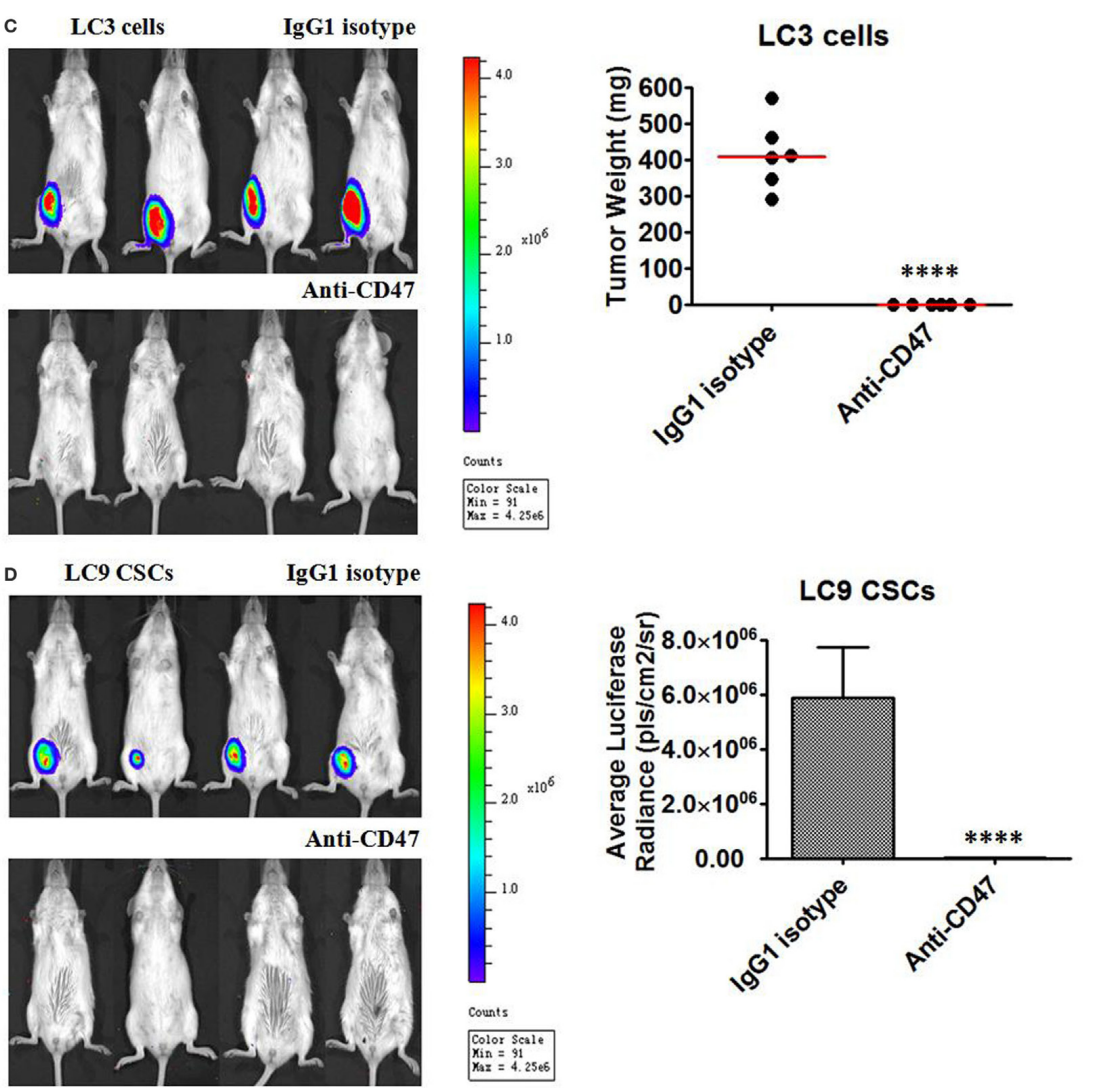
***Ex vivo* coating of lung cancer cells and lung cancer stem cells (CSCs) with an anti-CD47 antibody inhibits tumor engraftment**. **(A–D)** Luciferase-expressing lung cancer cells [A549 cell line cells and primary LC3 tumor cells from a *de novo* small cell lung carcinoma (SCLC) patient of phase IIB] and lung CSCs [NCI-H520 cell line CSCs and primary LC9 CSCs from a *de novo* adenocarcinoma patient of phase IIIA] were precoated with IgG1 isotype control antibody or anti-human CD47 B6H12.2 antibody *in vitro*. NOD/SCID mice transplanted with the A549 cells **(A)**, NCI-H520 CSCs **(B)**, primary LC3 cells **(C)**, or primary LC9 CSCs **(D)** were subject to bioluminescent imaging. Bioluminescence for A549 cells, NCI-H520 CSCs, LC3 cells, and LC9 CSCs engrafted mice was quantified (*n* = 6 per antibody condition). No tumor engraftment was observed in mice transplanted with anti-CD47-coated cells, in contrast to the 100% engraftment with IgG-coated cells (*P* < 0.0001), for all tested cells including A549 cells **(A)**, NCI-H520 CSCs **(B)**, LC3 cells **(C)**, and LC9 CSCs **(D)**. Data are represented as mean ± SD.

Next, we detected the ability of anti-CD47-blocking antibody to eliminate lung cancer cells and lung CSCs *in vivo* by two different treatment strategies. First, the effect of *ex vivo* anti-CD47 antibody coating on the engraftment of human lung cancer cells and lung CSCs was tested. Luciferase-expressing A549 cell line was precoated *ex vivo* with anti-CD47 or IgG1 isotype antibody and transplanted subcutaneously into NOD/SCID mice. After 8 weeks, all (six of six) mice in control IgG treatment group developed large tumors, but no tumors were detected in the mice treated with anti-human CD47 antibody (Figure 4A; Figure S4A in Supplementary Material). We repeated the *in vivo* engraftment experiment with CSCs isolated from lung cancer cell line. FACS-purified luciferase-expressing NCI-H520 CSCs were precoated *ex vivo* with anti-CD47 or IgG1 isotype antibody and injected subcutaneously into NOD/SCID mice. After 12 weeks, all (six of six) mice in the control IgG treatment group developed large tumors, whereas no tumors were detected in the anti-human CD47 antibody-treated mice as evidenced by bioluminescence imaging (Figure 4B). In addition to these cell lines, we also examined patient-derived primary lung tumor cells for their ability to engraft NOD/SCID mice. Similar to the results with the cell lines, *ex vivo* coating of these primary tumor cells (Figure 4C) and primary CSCs (Figure 4D; Figure S4B in Supplementary Material) with anti-CD47 antibody, but not controls, resulted in complete inhibition of subcutaneous engraftment.

We analyzed ESA^+^ CD133^+^ cells in primary tumor cells and showed that there were 0.5–11.6% ESA^+^ CD133^+^ cells in the primary tumor cells (median 4.6%). There were more than 95% ESA^+^ CD133^−^ non-CSCs in tumors and ESA^+^ CD133^+^ CSCs were no more than 5%. Therefore, the antitumor effect obtained in the xenograft model may be mediated majorly by anti-CD47 antibody targeting ESA^+^ CD133^−^ tumor cells.

### Therapy with Anti-CD47 Antibody Eliminates Lung Cancer Cells and Lung CSCs in the Xenotransplant Models

**Figure 5:**
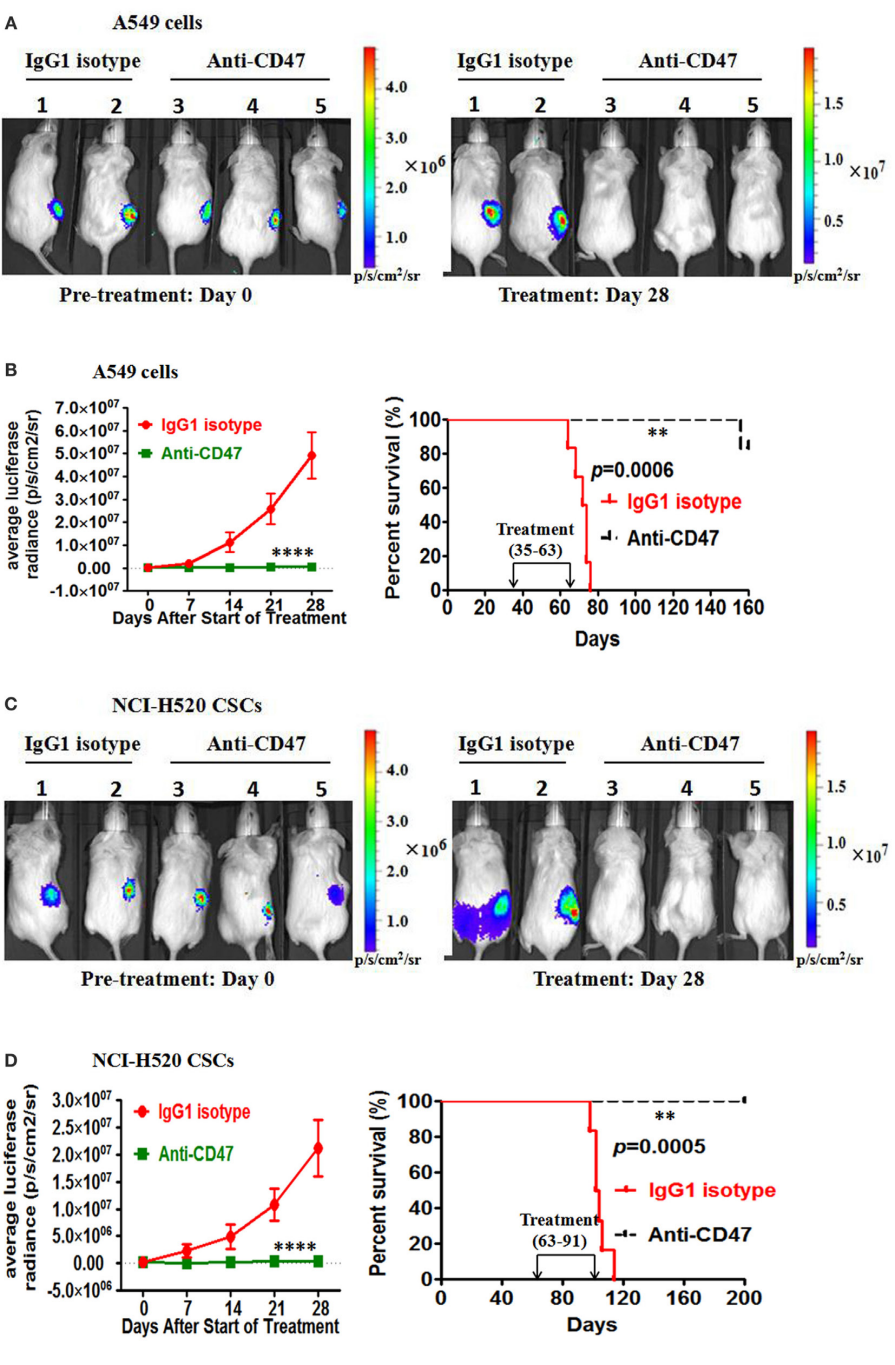

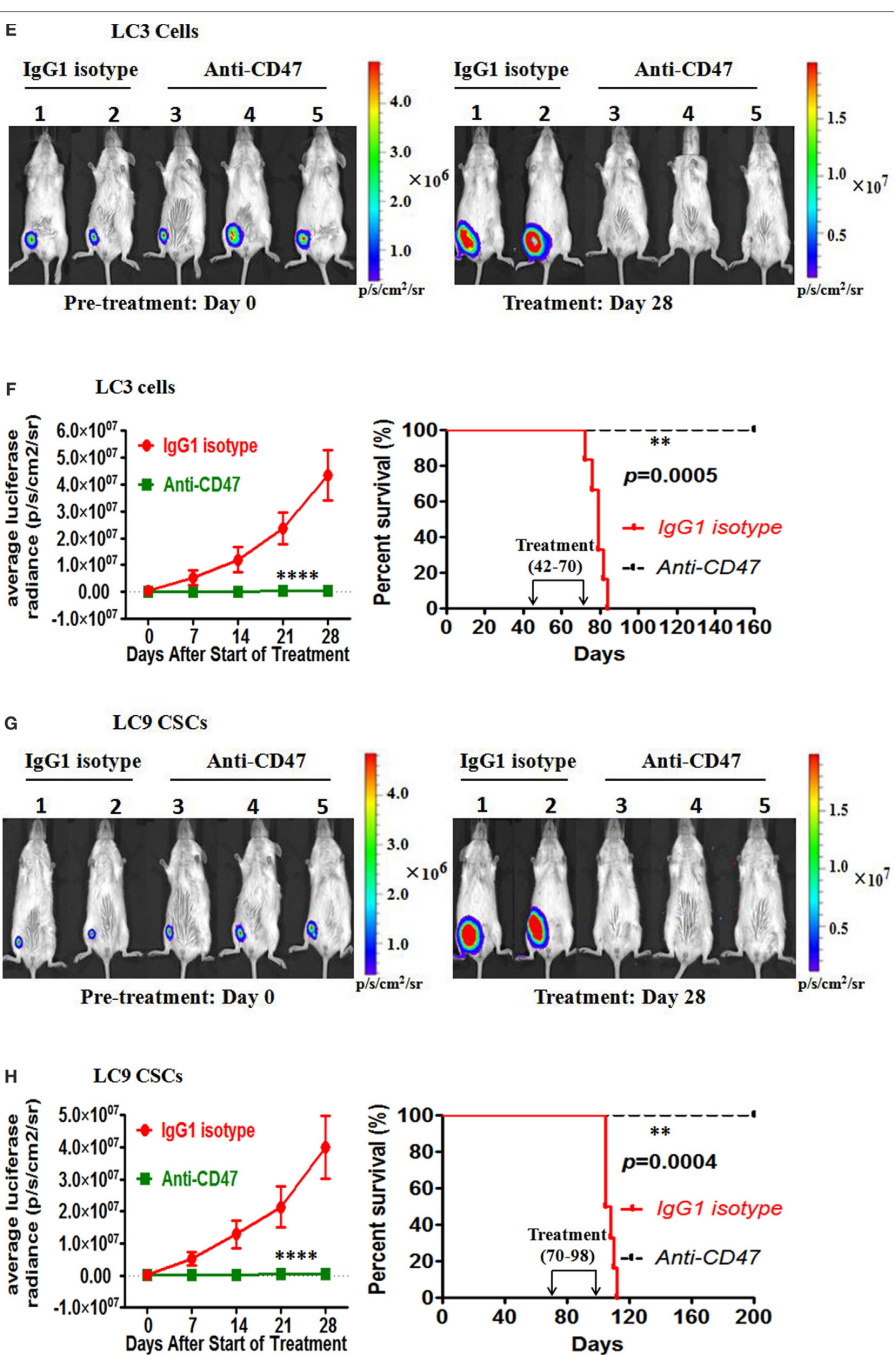
**Therapy with anti-CD47 antibody eliminates lung cancer cells and lung cancer stem cells (CSCs) in xenotransplant models**. **(A)** Luciferase-expressing A549 cells were transplanted subcutaneously into NOD/SCID mice. When palpable tumors (~100 mm^3^) formed, mice started to be treated with the IgG1 isotype antibody or anti-CD47 antibody (*n* = 6 per treatment group). Luciferase imaging of representative mice from each treatment group was shown before (day 0) and during (day 28) treatment **(A)**. Average bioluminescence and improved survival were shown **(B)**. **(C)** NOD/SCID mice were transplanted subcutaneously with luciferase-expressing NCI-H520 CSCs. When palpable tumors (~100 mm^3^) formed, mice started to be treated with the IgG1 isotype antibody or anti-CD47 antibody (*n* = 6 per treatment group). Luciferase imaging of representative mice from pretreatment and 28 days posttreatment were shown **(C)**. Average bioluminescence and improved survival were shown **(D)**. **(E)** NOD/SCID mice were transplanted subcutaneously with luciferase-expressing primary LC3 tumor cells. When palpable tumors (~100 mm^3^) formed, mice started to be treated with the IgG1 isotype antibody or anti-CD47 antibody (*n* = 6 per treatment group). Luciferase imaging of representative mice from pretreatment and 28 days posttreatment were shown **(E)**. Average bioluminescence and improved survival were shown **(F)**. **(G)** Luciferase-expressing primary LC9 CSCs were transplanted subcutaneously into the NOD/SCID mice. When palpable tumors (~100 mm^3^) formed, mice started to be treated with the IgG1 isotype antibody or anti-CD47 antibody (*n* = 6 per treatment group). Luciferase imaging of representative mice from pretreatment and 28 days posttreatment were shown **(G)**. Average bioluminescence and improved survival were shown **(H)**. ****P < 0.0001.

In the second treatment strategy, mice were first engrafted with lung cancer cells or lung CSCs and then administered anti-CD47 B6H12.2 antibody therapy. First, luciferase-expressing A549 cells were transplanted subcutaneously into NOD/SCID mice. Five weeks later, these mice were administered daily by intraperitoneal injections with either IgG1 isotype antibody or anti-human CD47 B6H12.2 antibody. Anti-CD47 antibody treatment decreased the tumor burden in these mice and significantly prolonged survival compared to control IgG1 (Figures 5A,B). Next, we purified the CSCs from the luciferase-expressing NCI-H520 cell line by FACS. Luciferase-expressing NCI-H520 CSCs were transplanted subcutaneously into NOD/SCID mice. Twelve weeks later, these mice were administered daily with either IgG1 isotype antibody or anti-human CD47 B6H12.2 antibody. Treatment with anti-human CD47 antibody inhibited CSC-initiated tumor growth as evidenced by bioluminescence imaging. Importantly, the mice treated with anti-CD47 indicated a dramatic increase in survival (Figures 5C,D). We also repeated the *in vivo* engraftment experiments with primary lung cancer cells and primary lung CSCs. In these two additional xenotransplantation models, anti-CD47 antibody treatment eliminated all of the primary lung cancer cells (Figures 5E,F) and primary lung CSCs (Figures 5G,H) in mice and significantly prolonged survival compared to IgG control (Figures 5E–H).

We further tested whether anti-human CD47 antibody can increase the number of macrophages in the tumor tissues in the xenotransplant models. Primary LC12 cells were subcutaneously transplanted into the NOD/SCID mice. When the sizes of the tumors reached ~200 mm^3^ after 6 weeks, 400 µg IgG1 isotype antibody or anti-human CD47 B6H12.2 antibody was intraperitoneally injected into the mice. After 48 h, the tumors were collected from the mice and disassociated for FACS (*n* = 4 per antibody condition). The results indicated that numbers of macrophages in the tumor tissues in anti-CD47-treated mice were significantly higher compared to the IgG1 isotype-treated control mice (Figure S5 in Supplementary Material). These data collectively indicate that anti-CD47 antibodies can recruit macrophages into the tumor microenvironment, dramatically inhibiting the growth of lung cancer cells and eliminated lung CSCs, thereby blocking the ability of CD47 to transmit the “don’t-eat-me” signal to macrophages.

### Anti-CD47 Antibody Exhibited No Significant Toxic Effect except Temporary White Blood Cell Reduction and Anemia

**Figure 6:**
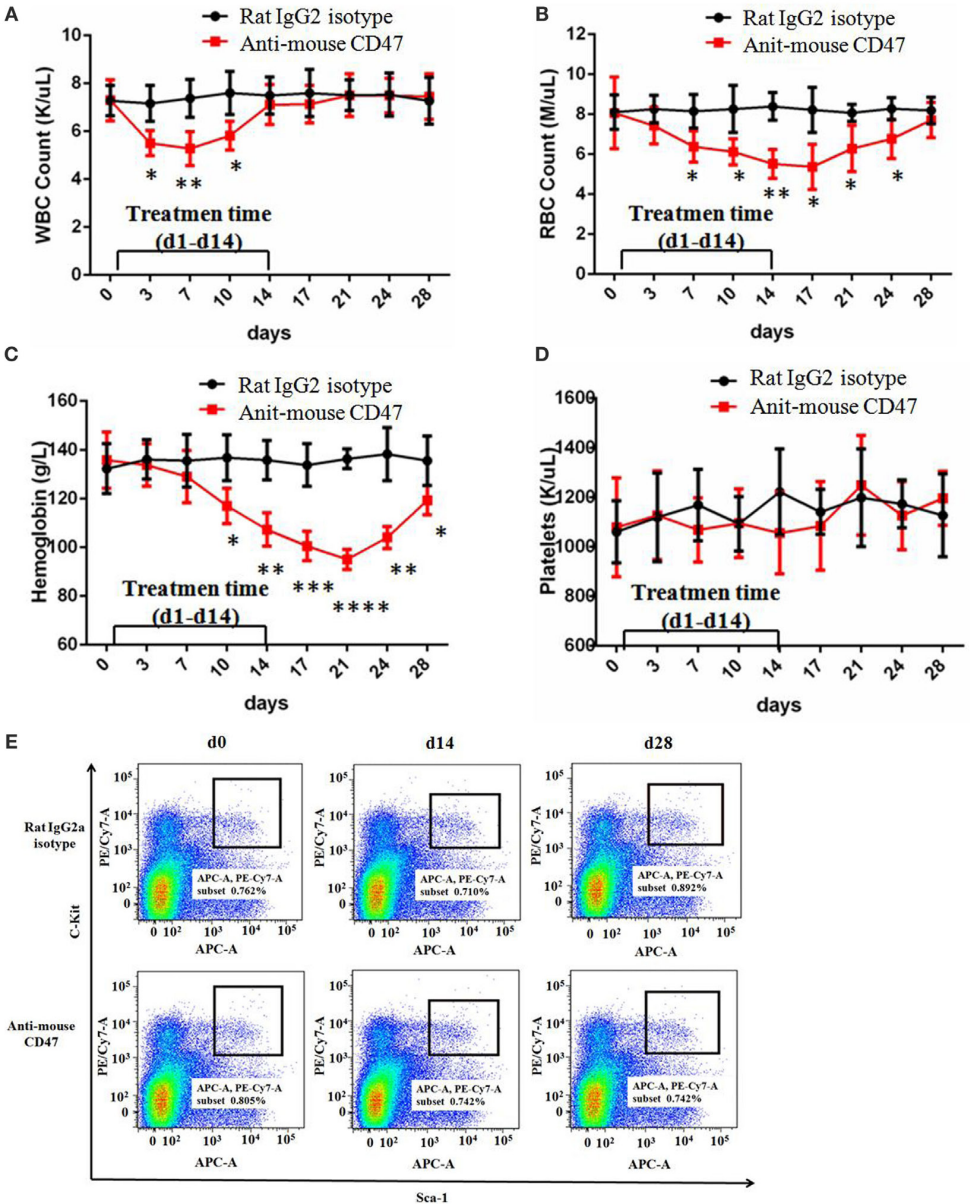

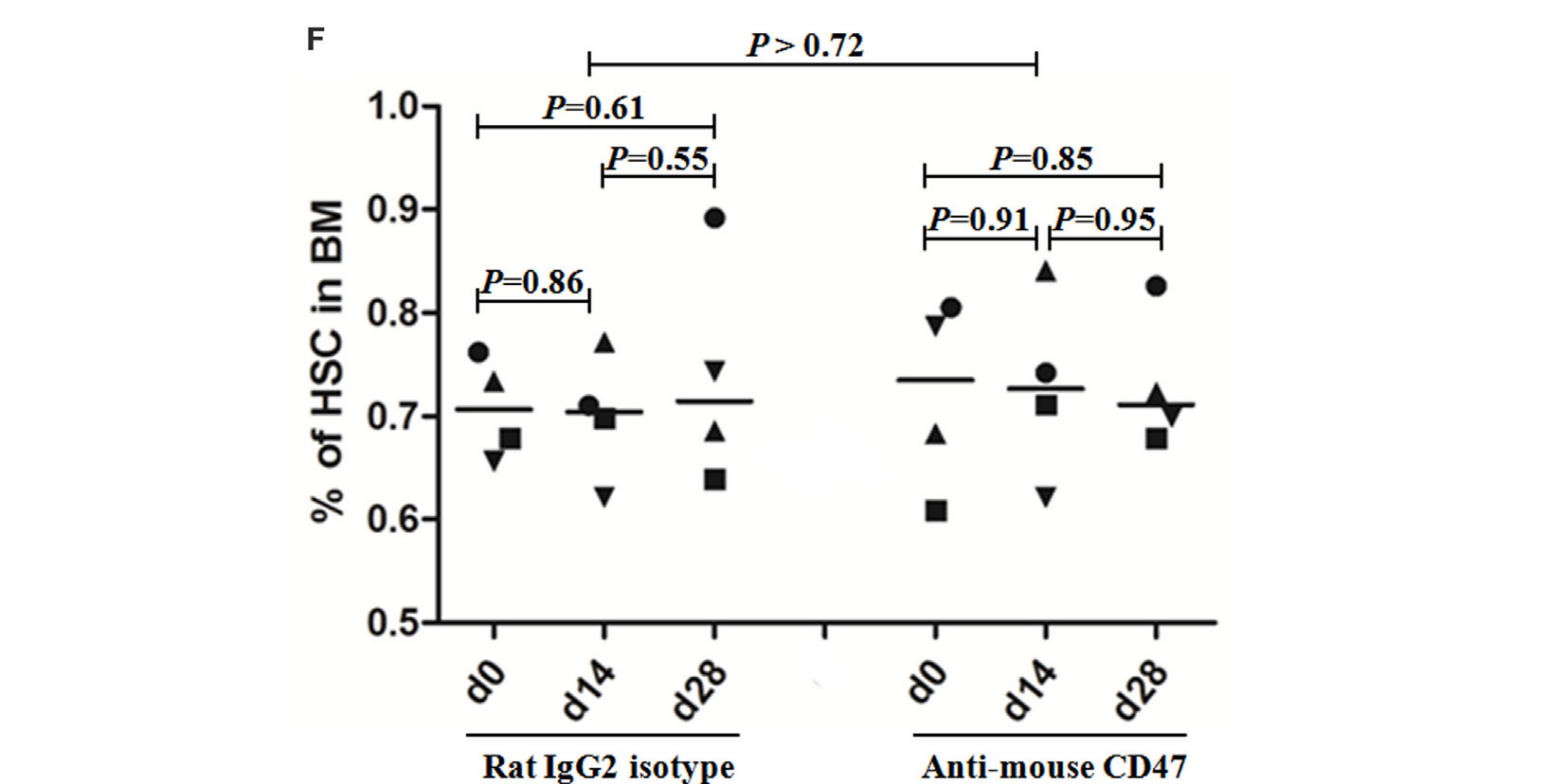
**Anti-CD47 antibody exhibited no significant toxic effect except temporary white blood cell reduction and anemia**. **(A–D)** Rat IgG2 isotype antibody or anti-mouse CD47 MIAP301 antibody was intraperitoneally injected into normal C57BL/6 mice at the dose of 400 µg daily from day 1 to day 14 (*n* = 4 per treatment group). These mice were followed up till day 28. White blood cell count **(A)**, red blood cell count **(B)**, and hemoglobin level **(C)** were temporarily decreased in anti-mouse CD47 group compared to IgG2 isotype group. Platelets count **(D)** has no difference between the two groups. **(E–F)** Mouse models were created as **(A–D)**. Bone marrow from these mice was aspirated at day 0, day 14, and day 28 and indicated no effect of treatment on the frequency of Lin^−^Kit^+^Sca^+^ hematopoietic stem cell (HSC) in the bone marrow. **(E)** Representative FACS plots are shown. **(F)** No differences in the percentage of HSC at day 0, day 14, and day 28 were observed with either control IgG or anti-mouse CD47.

CD47 is expressed at low levels on most normal tissues, including HSC. To investigate the feasibility of targeting CD47 as a therapeutic strategy, we utilized a normal C57BL/6 mouse model to examine an anti-mouse CD47-blocking monoclonal antibody (MIAP301). Wild-type C57BL/6 mice were administered daily by intraperitoneal injections with 400 µg of anti-mouse CD47 antibody for 14 days and follow-up for 28 days. Complete blood counts showed that white blood cell count, red blood cell count, and hemoglobin level were temporarily decreased in the anti-CD47 antibody-treated mice (Figures 6A–C). There was no evidence of thrombocytopenia (Figure 6D). In another experiment using normal C57BL/6 mouse models established as above, mice were sacrificed on day 0, day 14, and day 28. Bone marrow analysis showed no difference in total cellularity (data not shown) and percentage of Lin^−^Kit^+^Sca^+^ HSCs (Figures 6E,F). H&E staining of mouse tissues showed no pathological evidence of lung, liver, brain, spleen, or kidney damage (Figure S6 in Supplementary Material). These results suggest that targeting CD47 with a blocking monoclonal antibody generates no significant toxicity and is a viable therapeutic strategy.

## Discussion

CD47 is involved in the regulation of macrophages’ phagocytosis to prevent host cells from being eliminated (29). The mechanism of CD47-regulating phagocytosis is through its interaction with the protein receptor SIRPα on macrophages (17, 30, 31). HSCs and LSCs escape from macrophage’s phagocytosis through upregulating CD47 expression level (21, 22). Hence, CD47 serves as a target in the treatment of acute mylocytic leukemia (AML) by utilizing antibodies against CD47 to facilitate AML elimination by macrophages, which results in the blockade of inhibitory CD47–SIRPα signaling (21, 22). Thus far, CD47 has been found to be expressed on multiple human tumors, and anti-CD47 antibody can inhibit growth of these tumors *in vitro* and *in vivo* (23, 26, 32–38). CD47 blockade is considered as another immune checkpoint therapy for cancer (39). However, it is unclear how CD47 is involved in lung cancer initiation and progression. In this study, we indicate that higher expression of CD47 is also the mechanism used by lung cancer cells, especially lung CSCs, to escape phagocytosis. Blocking antibodies that dismiss the interaction between CD47 and SIRPα enable the phagocytosis of lung cancer cells and CSCs *in vitro* and inhibited tumor growth in several xenotransplantation models. In addition, the identification of *CD47* and *CD133* levels as prognostic factors could be incorporated into standard clinical prognostic considerations across multiple subtypes of lung cancer and may be useful in risk-adapted therapy decision-making. These results establish CD47 as a therapeutic target in lung cancer, as well as a therapeutic target in lung CSCs.

The toxicity of anti-CD47 antibody should be concerned when we translate this therapy to human application. CD47 is expressed on normal (non-tumor) cells at varying levels, but highly on tumor cells (16). In our study, we show that normal C57BL/6 mice treated with therapeutic doses of anti-mouse CD47-blocking antibody had no significant toxic effect except temporary anemia and white blood cells reduction (Figure 6; Figure S6 in Supplementary Material). The similar results were obtained in previous studies (22, 26). In these reports, mice were administered with 200–400 µg doses of anti-CD47 antibodies that may be in far excess of the minimal effective dose. Moreover, anti-CD47 antibody could not induce phagocytosis of non-cancer or normal cells *in vitro* (22, 27). These findings suggest that normal healthy cells are not subject to phagocytosis by phagocytes, because of lack a secondary prophagocytic “eat me” signaling, even deficiency of CD47–SIRPα signaling. Calreticulin and phosphatidylserine, as cell surface prophagocytic signals, have been identified (40, 41). Calreticulin can interact with LDL-receptor-related protein 1 on macrophages and is required for the phagocytosis of tumor cell lines, whose function is to neutralize CD47–SIRPα interaction (42). Calreticulin is expressed on cell surfaces of human leukemias, lymphomas, and solid cancers, but not on non-tumor or normal cell surfaces (38). Those prophagocytic signals on tumor cells may provide a therapeutic window to treatment with anti-CD47 antibodies without causing phagocytosis of normal cells that lack these “eat me” signals.

Here, we also detected the efficacy of anti-CD47 antibody to inhibit lung cancer cell growth as a monotherapy. In our study, anti-human CD47 antibody remarkably enhanced the phagocytosis of lung cancer cell lines and primary tumor cells *in vitro* (Figures 3A–C) and significantly inhibited tumor growth in lung cancer cell and primary tumor cell xenotransplantation models (Figures 5A,B,E,F). To investigate whether NK cells could mediate tumor elimination by anti-CD47 antibody *in vitro*, we utilized human NK cells (CD3^−^CD56^+^CD7^+^) as effectors. Anti-CD47 antibody did not induce increased ADCC of lung cancer cell line cells or primary patients tumor cells compared to IgG1 isotype control. However, anti-CD47 antibody caused increased ADCC of these cells compared to isotype control when we repeated these assays with mouse NK cells (CD3^−^DX5^+^) as effectors (Figures 3F,G). This indicated that NK cell was a potential contributor to tumor suppression in anti-CD47 antibody antitumor, and the antitumor effect obtained in the xenograft model may in part be mediated by mouse NK cells. In a previous study, anti-CD47 antibody can inhibit breast cancer, bladder cancer, ovarian cancer, colorectal cancer, and glioblastoma growth as a monotherapy (26). Together with our results and previous study, it suggested that CD47 is a promising therapeutic target in various types of solid tumor cells.

Cancer stem cells are a small subgroup of cancer cells that structure a reservoir of self-sustaining cells with the characteristic ability to self-renew and maintain the tumor mass (4, 43). The CSC model has been successfully used to explain events in tumor initiation, development, metastasis, relapse, and ineffectiveness of conventional cancer therapies. Conventional treatments directed against the bulk of the tumor cells may produce remarkable initial responses, but they are unlikely to result in long-term remissions if the rare CSCs are not targeted and escape (44). Here, we selected CD133 as the lung CSC marker (3–6) and purified CSCs from the luciferase-expressing NCI-H520 cell line and primary tumor cells by FACS. Then, we evaluated the efficacy of anti-CD47 antibody to inhibit lung CSCs as a monotherapy. In our study, anti-human CD47 antibody remarkably enhanced the phagocytosis of lung CSCs from the cell line and primary tumors *in vitro* (Figures 3D–F) and significantly inhibited tumor growth in the two lung CSCs xenotransplantation models (Figures 5C,D,G,H). In a previous report, anti-CD47 antibody can inhibit LSCs, breast CSCs, and bladder CSCs as a monotherapy (22, 26, 28). These results suggested that CD47 can be used as a therapeutic target to eliminate solid tumor CSCs.

In conclusion, we found that CD47 is expressed on lung cancer cells and lung CSCs, and *CD47* and *CD133* levels as prognostic factors can be useful in risk-adapted therapy decision-making of lung cancer patients. We have validated an important function of CD47 on lung cancer cells, especially lung CSCs, as a “don’t-eat-me” signal. We have also indicated that anti-CD47-blocking antibody is effective for treating human lung cancer and CSCs *in vitro* and *in vivo*. We anticipate that CD47 can be as a therapeutic target against solid tumors cells and their CSCs.

## Ethics Statement

Lung cancer tissue specimens were obtained from consented patients as approved by National Clinical Research Center of Cancer of China Review Board protocols. The experimental procedures involving human data were reviewed and approved by the Ethics Committee of National Clinical Research Center of Cancer of China. All subjects gave written informed consent in accordance with the Declaration of Helsinki. All experiments involving mice were performed according to Tianjin Medical University Cancer Institute and Hospital animal guidelines. The experimental protocol involving animals were reviewed and approved by the Ethics committee of Tianjin Medical University Cancer Institute and Hospital.

## Author Contributions

LL, LZ, HL, and XR designed the experiments. LL, SW, and XR wrote the manuscript. LL, LZ, LY (Lin Yang), JY, LY (Lili Yang), CY, FW, QS, RL, and HZ performed the experiments and analyzed data. LL, FY, HJ, JW, and LZ provided patient samples and clinical data.

## Disclaimer

This study has not been presented in part anywhere.

## Conflict of Interest Statement

The authors declare that the research was conducted in the absence of any commercial or financial relationships that could be construed as a potential conflict of interest.

## References

[1] Jordan CT Guzman ML Noble M Cancer stem cells. N Engl J Med (2006) 355:1253–61. 10.1056/NEJMra061808 16990388

[2] Reya T Morrison SJ Clarke MF Weissman IL. Stem cells, cancer, and cancer stem cells. Nature (2001) 414:105–11. 10.1038/35102167 11689955

[3] Pine SR Ryan BM Varticovski L Robles AI Harris CC. Microenvironmental modulation of asymmetric cell division in human lung cancer cells. Proc Natl Acad Sci U S A (2010) 107:2195–200. 10.1073/pnas.0909390107 20080668 PMC2836660

[4] O’Brien CA Kreso A Jamieson CH. Cancer stem cells and self-renewal. Clin Cancer Res (2010) 16:3113–20. 10.1158/1078-0432.CCR-09-2824 20530701

[5] Bertolini G Roz L Perego P Tortoreto M Fontanella E Gatti L Highly tumorigenic lung cancer CD133+ cells display stem-like features and are spared by cisplatin treatment. Proc Natl Acad Sci U S A (2009) 106:16281–6. 10.1073/pnas.0905653106 19805294 PMC2741477

[6] Eramo A Lotti F Sette G Pilozzi E Biffoni M Di Virgilio A Identification and expansion of the tumorigenic lung cancer stem cell population. Cell Death Differ (2008) 15:504–14. 10.1038/sj.cdd.4402283 18049477

[7] Ginestier C Hur MH Charafe-Jauffret E Monville F Dutcher J Brown M ALDH1 is a marker of normal and malignant human mammary stem cells and a predictor of poor clinical outcome. Cell Stem Cell (2007) 1:555–67. 10.1016/j.stem.2007.08.014 18371393 PMC2423808

[8] Al-Hajj M Wicha MS Benito-Hernandez A Morrison SJ Clarke MF. Prospective identification of tumorigenic breast cancer cells. Proc Natl Acad Sci U S A (2003) 100:3983–8. 10.1073/pnas.0530291100 12629218 PMC153034

[9] Singh SK Hawkins C Clarke ID Squire JA Bayani J Hide T Identification of human brain tumour initiating cells. Nature (2004) 432:396–401. 10.1038/nature03128 15549107

[10] Ricci-Vitiani L Lombardi DG Pilozzi E Biffoni M Todaro M Peschle C Identification and expansion of human colon-cancer-initiating cells. Nature (2007) 445:111–5. 10.1038/nature05384 17122771

[11] O’Brien CA Pollett A Gallinger S Dick JE A human colon cancer cell capable of initiating tumour growth in immunodeficient mice. Nature (2007) 445:106–10. 10.1038/nature05372 17122772

[12] Prince ME Sivanandan R Kaczorowski A Wolf GT Kaplan MJ Dalerba P Identification of a subpopulation of cells with cancer stem cell properties in head and neck squamous cell carcinoma. Proc Natl Acad Sci U S A (2007) 104:973–8. 10.1073/pnas.0610117104 17210912 PMC1783424

[13] Li C Heidt DG Dalerba P Burant CF Zhang L Adsay V Identification of pancreatic cancer stem cells. Cancer Res (2007) 67:1030–7. 10.1158/0008-5472.CAN-06-2030 17283135

[14] Patrawala L Calhoun T Schneider-Broussard R Li H Bhatia B Tang S Highly purified CD44+ prostate cancer cells from xenograft human tumors are enriched in tumorigenic and metastatic progenitor cells. Oncogene (2006) 25:1696–708. 10.1038/sj.onc.1209327 16449977

[15] Schatton T Murphy GF Frank NY Yamaura K Waaga-Gasser AM Gasser M Identification of cells initiating human melanomas. Nature (2008) 451:345–9. 10.1038/nature06489 18202660 PMC3660705

[16] Brown EJ Frazier WA Integrin-associated protein (CD47) and its ligands. Trends Cell Biol (2001) 11:130–5. 10.1016/S0962-8924(00)01906-1 11306274

[17] Jiang P Lagenaur CF Narayanan V. Integrin-associated protein is a ligand for the P84 neural adhesion molecule. J Biol Chem (1999) 274:559–62. 10.1074/jbc.274.2.559 9872987

[18] Oldenborg PA. Role of CD47 in erythroid cells and in autoimmunity. Leuk Lymphoma (2004) 45:1319–27. 10.1080/1042819042000201989 15359629

[19] Oldenborg PA Gresham HD Lindberg FP. CD47-signal regulatory protein alpha (SIRPalpha) regulates Fcgamma and complement receptor-mediated phagocytosis. J Exp Med (2001) 193:855–62. 10.1084/jem.193.7.855 11283158 PMC2193364

[20] Blazar BR Lindberg FP Ingulli E Panoskaltsis-Mortari A Oldenborg PA Iizuka K CD47 (integrin-associated protein) engagement of dendritic cell and macrophage counterreceptors is required to prevent the clearance of donor lymphohematopoietic cells. J Exp Med (2001) 194:541–9. 10.1084/jem.194.4.541 11514609 PMC2193501

[21] Jaiswal S Jamieson CH Pang WW Park CY Chao MP Majeti R CD47 is upregulated on circulating hematopoietic stem cells and leukemia cells to avoid phagocytosis. Cell (2009) 138:271–85. 10.1016/j.cell.2009.05.046 19632178 PMC2775564

[22] Majeti R Chao MP Alizadeh AA Pang WW Jaiswal S Gibbs KD Jr CD47 is an adverse prognostic factor and therapeutic antibody target on human acute myeloid leukemia stem cells. Cell (2009) 138:286–99. 10.1016/j.cell.2009.05.045 19632179 PMC2726837

[23] Krampitz GW George BM Willingham SB Volkmer JP Weiskopf K Jahchan N Identification of tumorigenic cells and therapeutic targets in pancreatic neuroendocrine tumors. Proc Natl Acad Sci U S A (2016) 113:4464–9. 10.1073/pnas.1600007113 27035983 PMC4843455

[24] McCracken MN Cha AC Weissman IL Molecular pathways: activating T cells after cancer cell phagocytosis from blockade of CD47 “don’t eat me” signals. Clin Cancer Res (2015) 21:3597–601. 10.1158/1078-0432.CCR-14-2520 26116271 PMC4621226

[25] Weiskopf K Ring AM Ho CC Volkmer JP Levin AM Volkmer AK Engineered SIRPα variants as immunotherapeutic adjuvants to anticancer antibodies. Science (2013) 341:88–91. 10.1126/science.1238856 23722425 PMC3810306

[26] Willingham SB Volkmer JP Gentles AJ Sahoo D Dalerba P Mitra SS The CD47-signal regulatory protein alpha (SIRPa) interaction is a therapeutic target for human solid tumors. Proc Natl Acad Sci U S A (2012) 109:6662–7. 10.1073/pnas.1121623109 22451913 PMC3340046

[27] Chao MP Alizadeh AA Tang C Myklebust JH Varghese B Gill S. Anti-CD47 antibody synergizes with rituximab to promote phagocytosis and eradicate non-Hodgkin lymphoma. Cell (2010) 142:699–713. 10.1016/j.cell.2010.07.044 20813259 PMC2943345

[28] Chan KS Espinosa I Chao M Wong D Ailles L Diehn M Identification, molecular characterization, clinical prognosis, and therapeutic targeting of human bladder tumor-initiating cells. Proc Natl Acad Sci U S A (2009) 106:14016–21. 10.1073/pnas.0906549106 19666525 PMC2720852

[29] Jaiswal S Chao MP Majeti R Weissman IL Macrophages as mediators of tumor immunosurveillance. Trends Immunol (2010) 31:212–9. 10.1016/j.it.2010.04.001 20452821 PMC3646798

[30] Seiffert M Brossart P Cant C Cella M Colonna M Brugger W Signal-regulatory protein alpha (SIRPalpha) but not SIRPbeta is involved in T-cell activation, binds to CD47 with high affinity, and is expressed on immature CD34(+)CD38(-) hematopoietic cells. Blood (2001) 97:2741–9. 10.1182/blood.V97.9.2741 11313266

[31] Barclay AN Brown MH. The SIRP family of receptors and immune regulation. Nat Rev Immunol (2006) 6:457–64. 10.1038/nri1859 16691243

[32] Chao MP Weissman IL Majeti R. The CD47-SIRPα pathway in cancer immune evasion and potential therapeutic implications. Curr Opin Immunol (2012) 24:225–32. 10.1016/j.coi.2012.01.010 22310103 PMC3319521

[33] Chao MP Tang C Pachynski RK Chin R Majeti R Weissman IL. Extranodal dissemination of non-Hodgkin lymphoma requires CD47 and is inhibited by anti-CD47 antibody therapy. Blood (2011) 118:4890–901. 10.1182/blood-2011-02-338020 21828138 PMC3208297

[34] Chao MP Alizadeh AA Tang C Jan M Weissman-Tsukamoto R Zhao F Therapeutic antibody targeting of CD47 eliminates human acute lymphoblastic leukemia. Cancer Res (2011) 71:1374–84. 10.1158/0008-5472.CAN-10-2238 21177380 PMC3041855

[35] Cioffi M Trabulo S Hidalgo M Costello E Greenhalf W Erkan M. Inhibition of CD47 effectively targets pancreatic cancer stem cells via dual mechanisms. Clin Cancer Res (2015) 21:2325–37. 10.1158/1078-0432.CCR-14-1399 25717063

[36] Steinert G Schölch S Niemietz T Iwata N García SA Behrens B Immune escape and survival mechanisms in circulating tumor cells of colorectal cancer. Cancer Res (2014) 74:1694–704. 10.1158/0008-5472.CAN-13-1885 24599131

[37] Pang WW Pluvinage JV Price EA Sridhar K Arber DA Greenberg PL Hematopoietic stem cell and progenitor cell mechanisms in myelodysplastic syndromes. Proc Natl Acad Sci U S A (2013) 110:3011–6. 10.1073/pnas.1222861110 23388639 PMC3581956

[38] Kaur S Elkahloun AG Singh SP Chen QR Meerzaman DM Song T A function-blocking CD47 antibody suppresses stem cell and EGF signaling in triple-negative breast cancer. Oncotarget (2016) 7:10133–52. 10.18632/oncotarget.7100 26840086 PMC4891109

[39] Vonderheide RH CD47 blockade as another immune checkpoint therapy for cancer. Nat Med (2015) 21:1122–3. 10.1038/nm.3965 26444633

[40] Ogden CA deCathelineau A Hoffmann PR Bratton D Ghebrehiwet B Fadok VA C1q and mannose binding lectin engagement of cell surface calreticulin and CD91 initiates macropinocytosis and uptake of apoptotic cells. J Exp Med (2001) 194:781–95. 10.1084/jem.194.6.781 11560994 PMC2195958

[41] Hoffmann PR deCathelineau AM Ogden CA Leverrier Y Bratton DL Daleke DL Phosphatidylserine (PS) induces PS receptor-mediated macropinocytosis and promotes clearance of apoptotic cells. J Cell Biol (2001) 155:649–59. 10.1083/jcb.200108080 11706053 PMC2198875

[42] Gardai SJ McPhillips KA Frasch SC Janssen WJ Starefeldt A Murphy-Ullrich JE Cell-surface calreticulin initiates clearance of viable or apoptotic cells through trans-activation of LRP on the phagocyte. Cell (2005) 123:321–34. 10.1016/j.cell.2005.08.032 16239148

[43] Clarke MF Dick JE Dirks PB Eaves CJ Jamieson CH Jones DL Cancer stem cells – perspectives on current status and future directions: AACR Workshop on cancer stem cells. Cancer Res (2006) 66:9339–44. 10.1158/0008-5472.CAN-06-3126 16990346

[44] Visvader JE. Cells of origin in cancer. Nature (2011) 469:314–22. 10.1038/nature09781 21248838

