# Supplementary material for: Anti-CD47 Antibody As a Targeted Therapeutic Agent for Human Lung Cancer and Cancer Stem Cells
Source: Front Immunol. 2017 Apr 21;8:404. doi: 10.3389/fimmu.2017.00404 (PMC5399041; doi:10.3389/fimmu.2017.00404)
Supplement: Supplementary file 1 [file Data_Sheet_1.PDF]

SUPPLEMENTAL MATERIALS

Supplementary Figures

Figure S1.

Sample	Age (Years)	Gender	WHO classification	Stage	Prior therapy	De Novo/ Relapsed
LC1	39	Male	AC	IIIA	None	De Novo
LC2	64	Female	SCC	IIA	None	De Novo
LC3	45	Male	SCLC	IIB	None	De Novo
LC4	51	Male	AC	IIA	None	De Novo
LC5	57	Female	SCC	IB	None	De Novo
LC6	61	Male	SCC	IIA	None	De Novo
LC7	48	Male	SCC	IIB	None	De Novo
LC8	71	Male	AC	IB	None	De Novo
LC9	52	Female	AC	IIIA	None	De Novo
LC10	55	Male	SCC	IIB	None	De Novo
LC11	43	Male	AC	IIB	None	De Novo
LC12	66	Male	SCC	IIIA	None	De Novo
LC13	69	Female	SCLC	IIB	None	De Novo
LC14	73	Male	AC	IIB	None	De Novo
LC15	46	Male	AC	IB	None	De Novo
LC16	61	Female	SCC	IIA	None	De Novo
LC17	68	Male	SCC	IIIA	None	De Novo
LC18	59	Male	SCC	IB	None	De Novo
LC19	47	Male	AC	IIB	None	De Novo
LC20	67	Female	AC	IIA	None	De Novo

Figure S2.

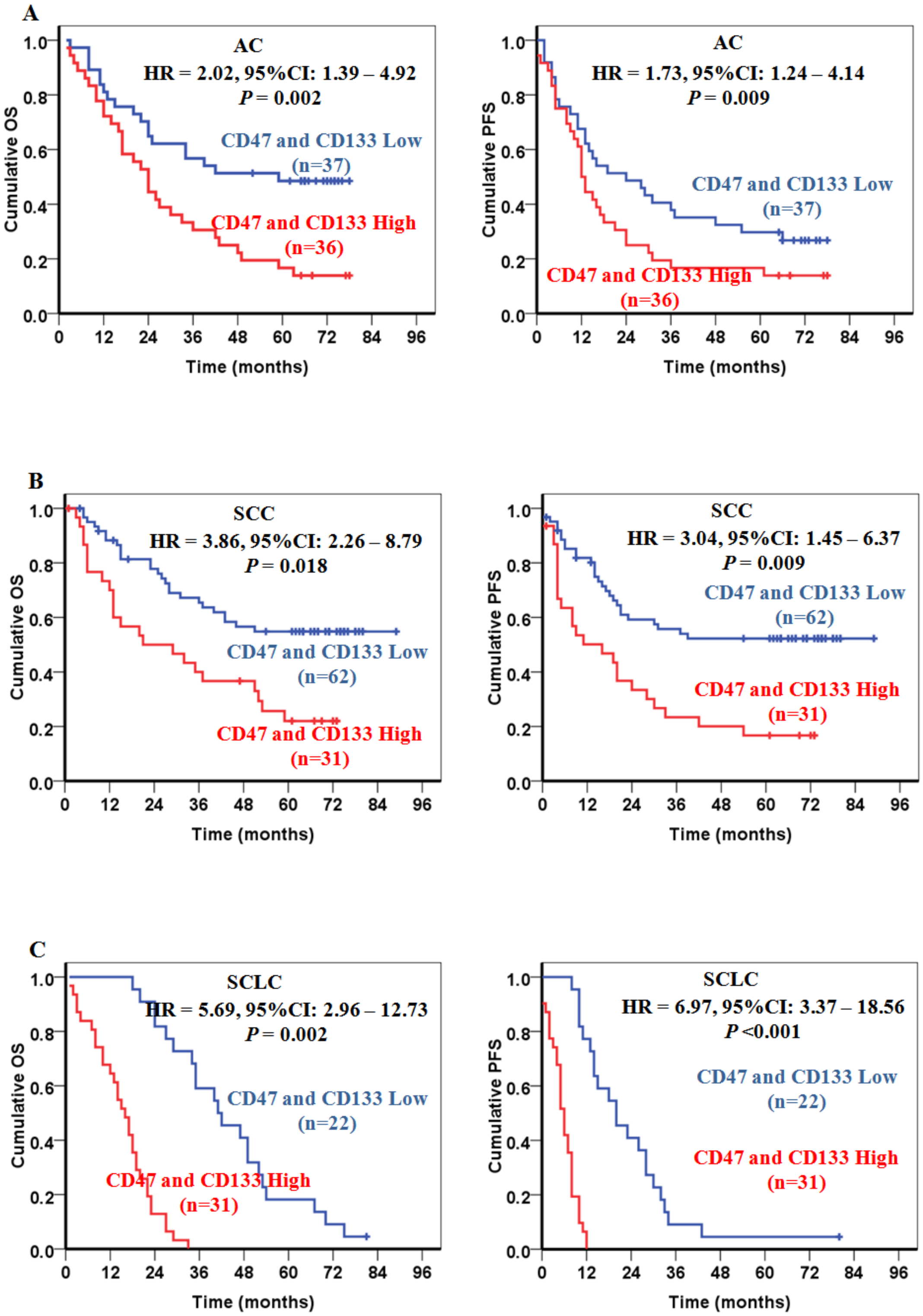
A
AC
HR = 2.02, 95%CI: 1.39 – 4.92
P = 0.002
CD47 and CD133 Low
(n=37)
CD47 and CD133 High
(n=36)
Cumulative OS
Time (months)
AC
HR = 1.73, 95%CI: 1.24 – 4.14
P = 0.009
CD47 and CD133 Low
(n=37)
CD47 and CD133 High
(n=36)
Cumulative PFS
Time (months)
B
SCC
HR = 3.86, 95%CI: 2.26 – 8.79
P = 0.018
CD47 and CD133 Low
(n=62)
CD47 and CD133 High
(n=31)
Cumulative OS
Time (months)
SCC
HR = 3.04, 95%CI: 1.45 – 6.37
P = 0.009
CD47 and CD133 Low
(n=62)
CD47 and CD133 High
(n=31)
Cumulative PFS
Time (months)
C
SCLC
HR = 5.69, 95%CI: 2.96 – 12.73
P = 0.002
CD47 and CD133 Low
(n=22)
CD47 and CD133 High
(n=31)
Cumulative OS
Time (months)
SCLC
HR = 6.97, 95%CI: 3.37 – 18.56
P <0.001
CD47 and CD133 Low
(n=22)
CD47 and CD133 High
(n=31)
Cumulative PFS
Time (months)

Figure S3.

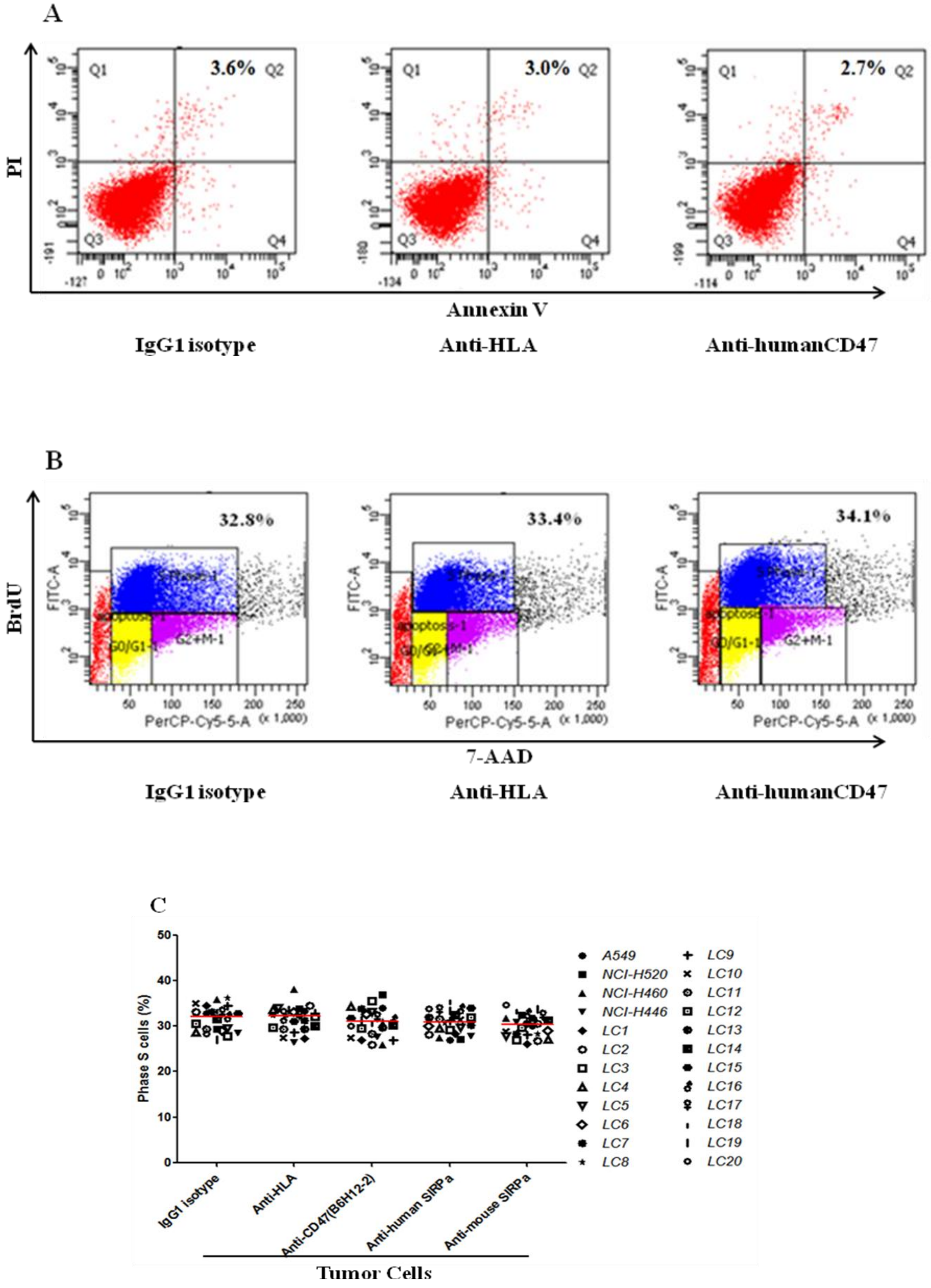
A
Q1
Q2
Q3
Q4
3.6%
3.0%
2.7%
PI
Annexin V
IgG1 isotype
Anti-HLA
Anti-humanCD47
B
32.8%
33.4%
34.1%
BrdU
FITC-A
PerCP-Cy5-5-A (x 1,000)
G0/G1-1
G2+M-1
apoptosis-1
7-AAD
IgG1 isotype
Anti-HLA
Anti-humanCD47
C
Phase S cells (%)
IgG1 isotype
Anti-HLA
Anti-CD47(B6H12-2)
Anti-human SIRPa
Anti-mouse SIRPa
Tumor Cells
A549
NCI-H520
NCI-H460
NCI-H446
LC1
LC2
LC3
LC4
LC5
LC6
LC7
LC8
LC9
LC10
LC11
LC12
LC13
LC14
LC15
LC16
LC17
LC18
LC19
LC20

Figure S4.

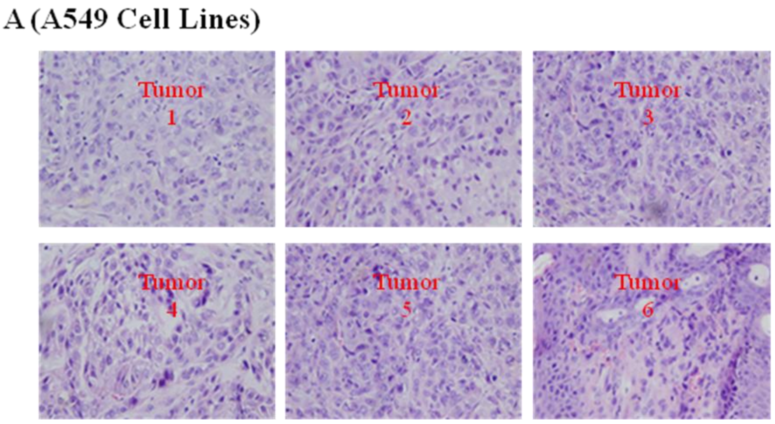
A (A549 Cell Lines)
Tumor 1
Tumor 2
Tumor 3
Tumor 4
Tumor 5
Tumor 6

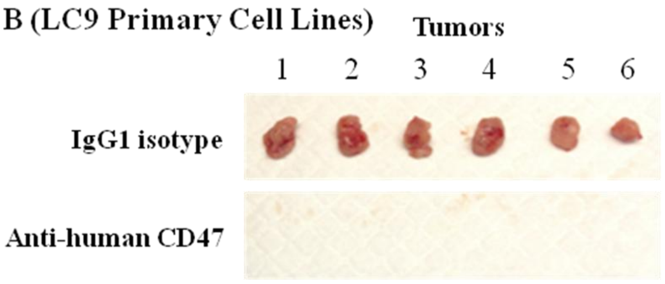
B (LC9 Primary Cell Lines)
Tumors
1 2 3 4 5 6
IgG1 isotype
Anti-human CD47

Figure S5.

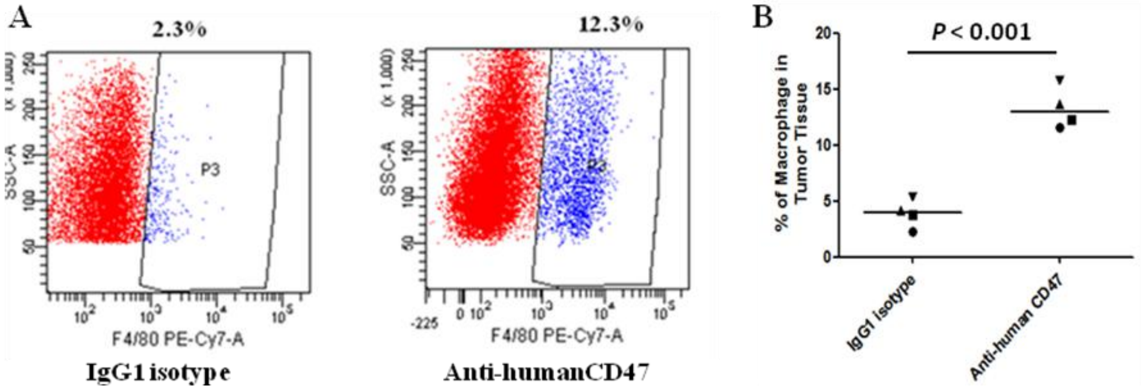
A
2.3%
12.3%
P3
SSC-A
(x 1,000)
F4/80 PE-Cy7-A
IgG1 isotype
Anti-humanCD47
B
P < 0.001
% of Macrophage in Tumor Tissue
IgG1 isotype
Anti-human CD47

Figure S6.

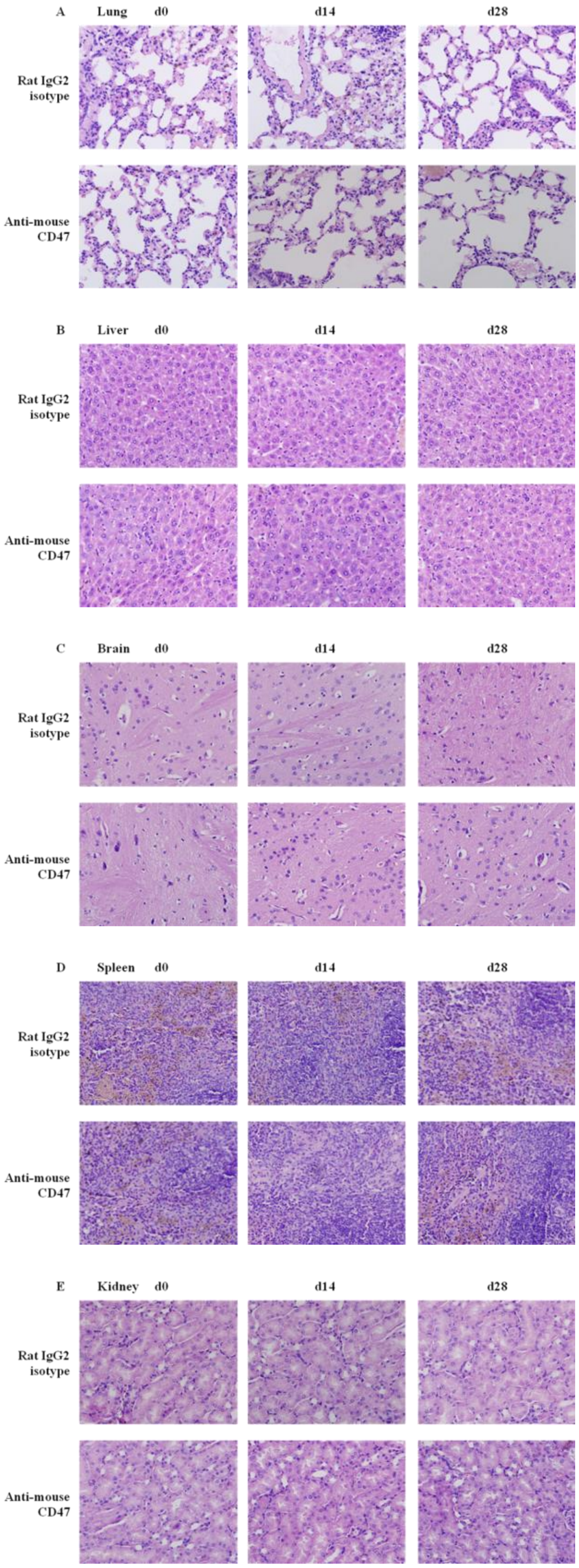
A
Lung
d0
d14
d28
Rat IgG2 isotype
Anti-mouse CD47
B
Liver
d0
d14
d28
Rat IgG2 isotype
Anti-mouse CD47
C
Brain
d0
d14
d28
Rat IgG2 isotype
Anti-mouse CD47
D
Spleen
d0
d14
d28
Rat IgG2 isotype
Anti-mouse CD47
E
Kidney
d0
d14
d28
Rat IgG2 isotype
Anti-mouse CD47

Supplemental Figure Legends

Supplementary Figure S1: Clinical parameters of primary human lung cancer samples used in vitro and/or in vivo. Stage is reported according to International Association for Study of Lung Cancer (IASLC) staging for all lung cancer patients.

Supplementary Figure S2: *CD47* and *CD133* mRNA double higher expression levels can decrease the outcome of lung cancer patients.

(A-C) In a multivariable analysis, *CD47* and *CD133* mRNA double higher expression levels were associated with a decreased probability of OS and PFS in patients with AC (A), SCC (B), and SCLC (C).

Supplementary Figure S3: Anti-human CD47 antibody-mediated effect on apoptosis and proliferation of lung cancer cells.

(A) Representative flow cytometry plots illustrating Annexin V/PI staining in primary LC4 cells treated with the indicated soluble antibodies. (B) Representative flow cytometry plots illustrating BrdU/7-AAD staining in primary LC12 cells treated with the indicated soluble antibodies. (C) Antibody-induced proliferation was tested by incubating lung cancer cells with the indicated antibodies and assessing the percentage of phase S cells. $P > 0.813$.

Supplementary Figure S4: Tumors in NOD/SCID mice transplanted with luciferase-expressing lung cancer cells.

(A) H&E staining of the six tumors in the six NOD/SCID mice transplanted with luciferase-expressing A549 cells precoated with IgG1 isotype antibodies. There was no tumor growth in NOD/SCID mice transplanted with luciferase-expressing A549 cells precoated with anti-human CD47 antibodies. (Related to Figure 4A) (B) Six tumors from the six NOD/SCID mice transplanted with luciferase-expressing primary LC9 cells precoated with IgG1 isotype antibodies. There was no tumor growth in NOD/SCID mice transplanted with luciferase-expressing primary LC9 cells precoated with anti-human CD47 antibodies. (Related to Figure 4D).

Supplementary Figure S5: Anti-human CD47 antibody increased the number of macrophages in xenografted tumors.

Primary LC12 cells were subcutaneously transplanted into NOD/SCID mice. When the size of the tumors reached 200mm^3 after 6 weeks, 400 μg IgG1 isotype antibody or anti-human CD47 antibody was intraperitoneally injected into the mice. After 48 hours, the tumors were collected from the mice, disassociated, and prepared for FACS analysis (n = 4 per antibody condition). (A) Representative FACS plots illustrating F4/80 staining in cells dissociated from the tumor tissues. (B) Anti-human CD47 antibody increased the number of macrophages in the tumor tissues compared to the IgG1 isotype control antibody.

Supplementary Figure S6: Administering therapeutic doses of blocking anti-mouse CD47 antibody MIAP301 to normal C57BL/6 mice exhibited no

significant toxic effect on mouse tissues (Related to Figure 6E).

(A-E) Representative H&E staining images of lung (A), liver (B), brain (C), spleen (D), and kidney (E) tissues.

Supplementary Tables

Table S1. Distributions of *CD47, CD133* mRNA relative expression levels in clinical characteristics of AC patients (n=100)

Clinical features		Median *CD47* expression levels (range)	*P* value		Median *CD133* expression levels (range)	*P* value
**Gender**			0.721			0.502
Male (n = 45)		1.431 (0.389-6.397)			2.178 (0.698-5.888)	
Female (n = 55)		1.501 (0.442-5.563)			2.000 (0.737-6.112)	
**Age (years)**			0.735			0.358
<60 (n = 47)		1.549 (0.499-5.563)			2.274 (0.985-5.322)	
≥60 (n = 53)		1.459 (0.389-6.397)			2.022 (0.698-6.112)	
**T phase**			0.123			0.274
T1+T2 (n = 75)		1.459 (0.389-6.397)			1.999 (0.698-6.112)	
T3+T4 (n = 25)		1.606 (0.600-4.875)			2.530 (1.218-4.105)	
**N phase**			**<0.001**			**0.017**
N0 (n = 51)		1.305 (0.442-4.372)			1.749 (0.701-6.112)	
N1+N2 (n = 49)		1.612 (0.389-6.397)			2.530 (0.698-5.036)	
**M phase**			**<0.001**			**0.002**
M0 (n = 81)		1.301 (0.389-4.876)			1.967 (0.698-6.112)	
M1+M2 (n = 19)		2.101 (0.512-6.397)			3.073 (0.987-5.487)	
**Clinical phase**			**<0.001**			**<0.001**
I-IIIA (n = 79)		1.399 (0.389-4.445)			1.557 (0.698-4.023)	
IIIB-IV (n = 21)		2.147 (1.301-6.397)			3.137 (2.137-6.112)	
**Smoking index**			0.246			**0.046**
≤0<400 (n = 60)		1.405 (0.449-6.397)			1.927 (0.265-5.021)	
≥400(n = 40)		1.522 (0.389-5.563)			2.675 (0.698-6.112)	
**KPS**			**<0.001**			**<0.001**
<80 (n = 39)		1.772 (0.884-6.397)			3.573 (1.781-5.012)	
≥80 (n = 61)		1.244 (0.389-4.378)			1.340 (0.698-6.112)	
**Hemoglobin**			0.087			0.912
<LLN (n = 4)		1.029 (0.703-4.301)			2.400 (1.016-4.772)	
≥LLN (n = 96)		1.500 (0.389-6.397)			2.105 (0.698-6.112)	
**White blood cells**			0.690			0.575
≤ULN (n = 93)		1.469 (0.389-6.397)			2.046 (0.698-4.116)	
>ULN (n = 7)		1.757 (0.669-4.903)			2.777 (1.037-6.112)	
**Platelets**			0.651			0.743
≤ULN (n = 86)		1.484 (0.389-6.397)			2.134 (0.698-6.112)	
>ULN (n = 14)		1.329 (0.600-4.173)			2.645 (1.037-4.577)	

Abbreviations: KPS, karnofsky performance status; LLN, lower limit of normal; ULN, upper limit of normal.

Table S2. Distributions of *CD47, CD133* mRNA relative expression levels in clinical characteristics of SCC patients (n = 147)

Clinical features		Median *CD47* expression levels (range)	*P* value		Median *CD133* expression levels (range)	*P* value
**Gender**			0.709			0.461
Male (n=120)		1.171 (0.113-6.049)			1.992 (0.265-5.423)	
Female (n = 27)		1.280 (0.216-5.136)			1.460 (0.511-5.834)	
**Age (years)**			0.221			0.134
<60 (n = 56)		1.238 (0.113-1.807)			2.247 (0.265-5.834)	
≥60 (n = 91)		1.158 (0.190-6.049)			1.790 (0.456-5.423)	
**T phase**			**0.043**			**0.023**
T1+T2 (n = 88)		1.132 (0.113-4.142)			1.490 (0.265-5.423)	
T3+T4 (n = 59)		1.300 (0.190-6.049)			2.303 (0.578-5.834)	
**N phase**			**0.001**			**0.003**
N0 (n = 86)		1.020 (0.113-4.781)			1.378 (0.265-4.912)	
N1+N2 (n = 61)		1.491 (0.190-6.049)			2.481 (0.511-5.834)	
**M phase**			**<0.001**			**0.011**
M0 (n = 132)		1.054 (0.113-6.049)			1.153 (0.265-5.834)	
M1+M2 (n = 15)		1.505 (0.200-5.649)			2.803 (0.532-5.423)	
**Clinical phase**			**<0.001**			**0.002**
I-IIIA (n = 125)		1.107 (0.113-6.049)			1.590 (0.265-5.011)	
IIIB-IV (n = 22)		1.573 (0.647-5.884)			2.870 (1.003-5.834)	
**Smoking index**			0.294			**0.934**
≤0<400 (n = 34)		1.262 (0.113-6.049)			1.893 (0.770-5.011)	
≥400 (n = 113)		1.306 (0.324-4.942)			1.994 (0.265-5.834)	
**KPS**			**0.001**			**0.001**
<80 (n = 53)		1.408 (0.355-4.884)			2.641 (0.610-5.834)	
≥80 (n = 94)		1.181 (0.113-6.049)			1.406 (0.265-5.307)	
**Hemoglobin**			0.072			0.210
<LLN (n = 16)		1.301 (0.418-4.703)			2.460 (0.785-5.132)	
≥LLN (n = 131)		1.262 (0.113-6.049)			1.945 (0.265-5.834)	
**White blood cells**			0.996			0.921
≤ULN (n = 124)		1.274 (0.200-6.049)			1.989 (0.456-5.834)	
>ULN (n = 23)		1.299 (0.113-5.684)			2.122 (0.265-4.132)	
**Platelets**			0.138			0.430
≤ULN (n = 97)		1.311 (0.200-6.049)			1.990 (0.532-5.834)	
>ULN (n = 50)		1.181 (0.113-5.751)			1.974 (0.265-4.226)	

Abbreviations: KPS, karnofsky performance status; LLN, lower limit of normal; ULN, upper limit of normal.

Table S3. Distributions of *CD47, CD133* mRNA relative expression levels in clinical characteristics of SCLC patients (n = 70)

Clinical features		Median *CD47* expression levels (range)	*P* value		Median *CD133* expression levels (range)	*P* value
**Gender**			0.976			0.642
Male (n=43)		2.417 (0.968-5.588)			2.325 (0.533-5.630)	
Female (n = 27)		2.435 (1.093-8.489)			2.211 (0.411-7.377)	
**Age (years)**			0.109			0.198
<60 (n = 29)		2.408 (0.974-5.588)			2.290 (0.411-5.542)	
≥60 (n = 41)		2.689 (0.968-8.489)			2.576 (1.135-7.377)	
**T phase**			0.117			0.124
T1+T2 (n = 54)		2.413 (0.968-4.972)			2.257 (0.411-6.113)	
T3+T4 (n = 16)		3.286 (1.261-8.489)			3.022 (0.876-7.377)	
**N phase**			**<0.001**			**<0.001**
N0 (n = 23)		1.910 (0.968-2.435)			1.489 (0.411-2.793)	
N1+N2 (n = 47)		2.691 (2.051-8.489)			2.477 (1.233-7.377)	
**M phase**			**<0.001**			**<0.001**
M0 (n = 25)		1.926 (0.968-2.508)			1.524 (0.411-4.386)	
M1+M2 (n = 45)		2.757 (1.903-8.489)			2.576 (1.233-7.377)	
**Clinical phase**			**<0.001**			**0.002**
I-IIIA (n = 23)		1.926 (0.968-2.499)			1.489 (0.411-2.450)	
IIIB-IV (n = 47)		2.691 (1.539-8.489)			2.587 (1.122-7.377)	
**Smoking index**			**0.003**			**0.001**
≤0<400 (n = 58)		2.370 (0.968-5.028)			2.205 (0.411-6.113)	
≥400 (n = 12)		4.092 (1.926-8.489)			3.895 (1.233-7.377)	
**KPS**			**<0.001**			**<0.001**
<80 (n = 47)		2.684 (1.808-8.489)			2.468 (1.169-7.377)	
≥80 (n = 23)		1.912 (0.968-3.899)			1.524 (0.411-4.386)	
**Hemoglobin**			0.112			0.069
<LLN (n = 6)		1.879 (0.968-8.489)			1.628 (1.122-4.132)	
≥LLN (n = 64)		2.447 (0.974-4.238)			2.321 (0.411-7.377)	
**White blood cells**			**0.050**			**0.045**
≤ULN (n = 67)		2.408 (0.968-8.489)			2.090 (0.411-7.377)	
>ULN (n = 3)		4.427 (2.757-4.929)			3.610 (2.895-4.132)	
**Platelets**			0.349			0.061
≤ULN (n = 54)		2.433 (0.968-5.588)			1.883 (0.411-7.377)	
>ULN (n = 16)		2.205 (0.974-8.489)			2.552 (0.685-6.113)	

Abbreviations: KPS, karnofsky performance status; LLN, lower limit of normal; ULN, upper limit of normal.

Table S4. Multivariable analysis of clinical characteristics and survival in 100 AC patients

Parameter	PFS			OS		
	HR	95% CI	*P* value	HR	95% CI	*P* value
CD47 mRNA expression levels	1.98	1.21-3.23	0.017	2.25	1.24-4.10	0.008
CD133 mRNA expression levels	1.79	1.09-2.92	0.035	1.77	1.02-3.48	0.038
Clinical phase	1.86	1.32-4.13	0.002	3.67	1.99-7.83	<0.001
No surgery	1.43	1.27-2.88	0.017	1.65	1.18-2.89	0.009
KPS≥80	0.31	0.15-0.76	0.025	0.25	0.13-0.57	0.003

Abbreviations: KPS, Karnofsky performance status; PFS, Progression-free survival; OS, Overall survival; HR, Hazard Ratio.

Table S5. Multivariable analysis of clinical characteristics and survival in 147 SCC patients

Parameter	PFS			OS		
	HR	95% CI	*P* value	HR	95% CI	*P* value
CD47 mRNA expression levels	1.21	1.01-2.45	0.039	1.25	1.08-3.42	0.025
CD133 mRNA expression levels	1.84	1.16-2.92	0.009	1.64	1.10-2.79	0.042
Clinical phase	2.33	1.34-4.05	0.003	2.99	1.52-4.99	<0.001
No surgery	1.62	1.21-3.69	0.022	1.71	1.02-2.86	0.038
Smoking index	1.51	1.06-2.39	0.048	2.48	1.51-4.06	0.008
KPS≥80	0.45	0.29-0.72	0.009	0.41	0.25-0.66	0.006

Abbreviations: KPS, Karnofsky performance status; PFS, Progression-free survival; OS, Overall survival; HR, Hazard Ratio.

Table S6. Multivariable analysis of clinical characteristics and survival in 70 SCLC patients

Parameter	PFS			OS		
	HR	95% CI	*P* value	HR	95% CI	*P* value
CD47 mRNA expression levels	3.31	1.45-8.51	0.002	3.52	1.73-7.17	0.001
CD133 mRNA expression levels	1.75	1.23-3.09	0.021	2.38	1.47-4.15	0.012
Clinical phase	1.95	1.52-2.51	<0.001	3.68	1.81-8.52	<0.001
No surgery	1.39	1.21-2.18	0.023	2.26	1.77-2.88	0.005
Smoking index	2.29	1.21-4.35	0.012	2.23	1.82-2.75	0.009
KPS≥80	0.47	0.22-0.95	0.036	0.33	0.15-0.75	0.007

Abbreviations: KPS, Karnofsky performance status; PFS, Progression-free survival; OS, Overall survival; HR, Hazard Ratio.
